# Efficacy and safety of danshen class injections in the treatment of coronary heart disease: a network meta-analysis

**DOI:** 10.3389/fphar.2024.1487119

**Published:** 2024-12-12

**Authors:** Shuang Dai, Yukun Ding, Jianbo Guo, Xian Wang

**Affiliations:** ^1^ Dongzhimen Hospital, Beijing University of Chinese Medicine, Beijing, China; ^2^ Science and Education Departmen, Beijing Fengtai Hospital of Integrated Traditional Chinese and Modern Medicine, Beijing, China; ^3^ LKS Faculty of Medicine, The University of Hong Kong, Pokfulam, Hong Kong SAR, China

**Keywords:** danshen class injections, Chinese medicine injection, coronary heart disease, network meta-analysis, randomized controlled trial

## Abstract

**Background:**

Danshen [Salvia miltiorrhiza Bunge (Lamiaceae; Salviae miltiorrhizae radix et rhizoma)] class injections (DSCIs) are widely used in the treatment of coronary heart disease (CHD). However, there are various types of DSCIs available on the market, and it remains uncertain which DSCI has the best clinical efficacy, as well as which one is most effective in regulating inflammatory markers and oxidative stress indicators. The aim of this network meta-analysis (NMA) is to compare the therapeutic effects of different DSCIs to identify the optimal DSCI for the treatment of CHD.

**Methods:**

The databases searched to identify randomized controlled trials (RCTs) of DSCIs for CHD included the China National Knowledge Infrastructure (CNKI), Wanfang Database, China Science and Technology Journal Database (VIP), Chinese Biomedical Literature Database (CBM), PubMed, Web of Science, and Cochrane Library. The search period spanned from the inception of each database up to June 2024. NMA was conducted using RevMan 5.3 and Stata 16.0 software.

**Results:**

A total of 106 studies including 14,979 patients, involving 10,931 patients, with 5,640 in the experimental group and 5,291 in the control group. And ten DSCIs were extracted, namely: Danhong injection (DH), Danshen injection (DS), Danshenchuanxiongqin injection (DSCXQ), Dansenduofensuanyan injection (DSDFSY), Danshenfen injection (DSFZ), Fufang Danshen injection (FFDS), Guanxinning injection (GXN), Sodium Tanshinone IIA Sulfonate injection (STS), Xiangdan injection (XD), Shenxiongputaotang injection (SXPTT). The results of NMA showed that, XD injection significantly enhances clinical efficacy; STS is more effective in reducing hs-CRP levels; DSDFSY shows better efficacy in decreasing IL-1 and increasing NO levels; DSCXQ has a greater advantage in reducing IL-6 levels; GXN is more effective in regulating SOD levels; and DH is better at reducing MDA levels.

**Conclusion:**

The combined treatment of DSCIs and WM more significant efficacy in patients with CHD compared to WM treatment alone, including clinical efficacy evaluation, inflammatory markers, and oxidative stress markers. Overall, DSDFSY and DSCXQ show better performance in clinical efficacy evaluation and regulation of inflammatory markers, while DH exhibits a more stable effect in regulating oxidative stress. However, larger sample sizes and high-quality RCTs are still necessary to further compare the various DSCIs.

**Systematic Review Registration:**

[PROSPERO], identifier [CRD42024548928].

## 1 Introduction

CHD is one of the most common cardiovascular diseases, with approximately 11.39 million patients in China (National Center for Cardiovascular Disease, 2023). According to the “China Health and Family Planning Statistical Yearbook 2021” (National Health Commission of the People’s Republic of China, 2021), the mortality rate of CHD in urban residents in China was 126.91/100,000 and 135.88/100,000 in rural areas in 2020, showing a continuous upward trend. Dyslipidemia (Martin et al., 2014) and inflammation (Hansson, 2005) jointly induce the formation of atherosclerotic plaques, which obstruct the coronary arteries and lead to CHD. CHD is a dynamic process characterized by the accumulation of atherosclerotic plaques and changes in coronary circulation. Clinically, CHD can be divided into chronic coronary syndrome (CCS) and acute coronary syndrome (ACS). The “2019 ESC Guidelines for the Diagnosis and Management of Chronic Coronary Syndromes” (Knuuti et al., 2020) recommend that conventional treatments for CCS include anti-ischemic therapy (Wight et al., 1992; Wei et al., 2011), antiplatelet therapy (Mehta et al., 2010), lipid-lowering therapy (Mach et al., 2020), and revascularization (Neumann et al., 2019). The “2023 ESC Guidelines for the Management of Acute Coronary Syndromes” (Byrne et al., 2023) suggest that the primary treatments for ACS should include percutaneous coronary intervention (PCI) (Huynh et al., 2009), thrombolytic therapy (Fibrinolytic Therapy Trialists FTT, 1994), and antithrombotic therapy (Valgimigli et al., 2018; Eikelboom et al., 2000). Additionally, anti-inflammatory and antioxidant treatments have been studied for decades in CHD patients (Malekmohammad et al., 2019), such as low-dose colchicine (Nidorf et al., 2020), IL-6 inhibitors (Broch et al., 2021), IL-1 inhibitors (Everett et al., 2020), vitamin C (Kaufmann et al., 2000), vitamin E (Riemersma, 1996), and β-carotene (Törnwall et al., 2004). Despite the gradual improvement of current treatment methods, challenges such as angina, antiplatelet drug resistance (Gorog et al., 2009), and microcirculation disorders (Padro et al., 2020) still exist. Therefore, comprehensively improving the quality of life for CHD patients remains a significant challenge.

CHD falls under the categories of “chest impediment and heart pain”, “sudden heart pain”, and “true heart pain” in traditional Chinese medicine (TCM). The earliest Chinese medicinal text, “Shen Nong’s Materia Medica,” documented that Danshen could treat evil qi in the heart and abdomen. In ancient China, decoctions and pills were the primary preparations of Danshen [Salvia miltiorrhiza Bunge (Lamiaceae; Salviae miltiorrhizae radix et rhizoma)], with the dried root being the most commonly used part. According to the “Chinese Pharmacopoeia” (2020), Danshen possesses effects such as promoting blood circulation, removing blood stasis, and cooling the blood. Modern research has identified various chemical metabolites in Danshen (Su et al., 2015), which can be categorized into three main types: diterpene quinones, including tanshinone-type diterpenes; hydrophilic phenolic acids, mainly phenolic acids; and essential oil metabolites, with diterpene quinones and hydrophilic phenolic acids being the primary active metabolites (Gao et al., 2012). Modern studies have discovered that Danshen has multiple pharmacological effects, including anti-myocardial ischemia, improvement of atherosclerosis, anti-inflammatory, antihypertensive, lipid-lowering, hypoglycemic, antithrombotic, and anti-tumor effects (Su et al., 2015). Therefore, DSCIs, which are TCM injections with Danshen as the main component, are widely used in treating CHD.

Previous studies have conducted meta-analyses on stable angina, unstable angina, or myocardial infarction (Zhang et al., 2017; Wu et al., 2017; Liu et al., 2018; Li et al., 2022), but no comprehensive meta-analysis on CHD as a whole has been done, and it remains unclear which injection is more effective for CHD. NMA was chosen for this study as it allows for the simultaneous comparison of multiple treatments, even when direct head-to-head trials are unavailable, by combining both direct and indirect evidence. This approach provides a more comprehensive and precise assessment of the relative efficacy of different DSCIs, offering insights that would be difficult to obtain through traditional Meta-Analysis alone. Therefore, we decided to use a network meta-analysis, incorporating both direct comparisons from RCTs and indirect comparisons based on shared control RCTs (Nikolakopoulou et al., 2018). We aim to identify the most reliable DSCIs for the treatment of CHD through relevant RCT-based network meta-analyses and to evaluate the relative efficacy and safety of different DSCIs in CHD patients, providing a reference for clinical application. This study focuses on patients with coronary heart disease (CHD), addressing a gap in previous research that has primarily focused on other conditions. We extended the search period to include studies from the past 2 years, enhancing the relevance and timeliness of our findings. Additionally, we explored changes in inflammatory markers and oxidative stress indicators, providing deeper insight into the underlying mechanisms of Danshen’s therapeutic effects. We also compared different Danshen injections, an area that has not been extensively addressed in previous meta-analyses, offering more precise conclusions about the relative efficacy of various formulations. These distinctive aspects highlight the originality and value of our study in advancing the understanding of Danshen’s role in treating CHD.

## 2 Methods

### 2.1 Study registering

The review protocol was registered at PROSPERO (No: CRD42024548928, https://www.crd.york.ac.uk/prospero/). The current research procedure was conducted following the guidelines of the Preferred Reporting Items for Systematic Reviews and Meta-Analyses (PRISMA) (Hutton et al., 2015).

### 2.2 Inclusion and exclusion criteria

The inclusion criteria for this study adhered to the PICOS framework, encompassing participants, interventions, comparisons, outcomes, and study design. Therefore, clinical trials meeting the following criteria were included:

(1) Participants: This study included patients diagnosed with CHD, including chronic coronary syndrome and acute coronary syndrome, without restrictions on race, gender, age, or nationality. Study design. Only RCTs mentioned in articles were enrolled.
(2) Interventions and comparisons: The experimental group received DSCIs combined with guideline-recommended Western medicine. Patients in the control group received Western medicine treatment alone, without the use of other Chinese medicine. Commonly used Western medicines included antiplatelet agents, statins, nitrates, β-receptor blockers, and Angiotensin-Converting Enzyme Inhibitors. There were no restrictions on dosage and duration of treatment. Appropriate treatment measures were taken for patients with additional comorbidities.
(3) Outcomes: The primary outcome of this study was the clinical effectiveness rate. Based on changes in clinical symptoms and objective indicators, the effectiveness status was categorized as effective or ineffective. When patients’ clinical symptoms showed no significant change or even worsened (e.g., increased frequency, longer duration, more intense pain), and there was no improvement in the electrocardiogram, it was considered ineffective. Secondary outcomes included high-sensitivity C-reactive protein (hs-CRP), interleukin-1 (IL-1), interleukin-6 (IL-6), nitric oxide (NO), superoxide dismutase (SOD), malondialdehyde (MDA), and adverse reactions. Included studies should have at least one outcome measure.
(4) Study design: Only Randomized Controlled Trial (RCT) were enrolled.

We excluded the following studies: (1) studies using other traditional Chinese medicine preparations or external traditional Chinese medicine therapies (such as massage, gua sha, cupping); (2) studies related to percutaneous coronary intervention (PCI) or thrombolytic surgery; (4) studies with incomplete or erroneous data; (5) studies for which the full text could not be obtained.

### 2.3 Search strategy

A systematic electronic search was conducted across eight databases to identify RCT studies published from their inception up to June 2024. The databases searched included the China National Knowledge Infrastructure Database (CNKI), the Chinese Scientific Journals Full-text Database (VIP), the Wan-Fang Database, the Chinese Biomedical Literature Database (SinoMed), the Cochrane Library, PubMed, Web of Science and Embase. The search was not restricted by language or country. For detailed search strategies, please refer to the appendix.

### 2.4 Data extraction

Two researchers (DS and DYK) used EndNote X9 for reference management, removing duplicate records and excluding irrelevant or non-compliant studies according to the inclusion and exclusion criteria. Subsequently, a database was established using Microsoft Excel to meticulously record study information, including publication details (title, author names, and publication date), patient information (sample size, mean age and gender composition, classification of coronary artery disease), interventions (name of the injection, dosage, and duration of administration), outcomes (primary and secondary outcomes), and study design (randomization, allocation concealment, and blinding). To ensure data accuracy, two independent researchers entered the data and cross-checked for inconsistencies. For studies with missing data, we contacted the original authors for clarification; if no accurate data was provided, the study was excluded from the analysis.

### 2.5 Quality assessment

Two researchers independently assessed the quality of all included studies according to the Cochrane Intervention Reviewer’s Handbook version 5.1.0 (Cumpston et al., 2019). The assessment criteria included random sequence generation (selection bias), allocation concealment (selection bias), blinding of participants and personnel (performance bias), blinding of outcome assessment (detection bias), incomplete outcome data (attrition bias), selective reporting (reporting bias), and other biases. Each criterion could be rated as low, high, or unclear risk of bias.

In cases of discrepancies in data extraction and quality assessment, resolution was achieved through the judgment of a third researcher or consensus.

### 2.6 Statistical analysis

Statistical and network meta-analysis were conducted using Stata 16.0 software. For dichotomous outcomes, results were presented as odds ratios (OR) with corresponding 95% confidence intervals (95% CI); for continuous variable outcomes, results were shown as mean differences (MD) with 95% CI. Additionally, if a particular outcome had two or more studies directly compared, a pairwise meta-analysis using a random-effects model was employed. Different interventions were compared through network meta-analysis using a frequentist framework and a random-effects model, and the results were presented in a ranking format. The surface under the cumulative ranking curve (SUCRA) was plotted according to the size of the cumulative ranking area under the curve, providing a more intuitive display of the ranking of each treatment measure. SUCRA ranges from 0% to 100%, assigned to the worst and best treatment measures respectively. Since no closed loops were formed in the analysis, inconsistency assessment was not feasible. Furthermore, publication bias of the included RCT was examined by comparing funnel plots corrected by regression lines.

## 3 Results

### 3.1 Literature selection

**FIGURE 1:**
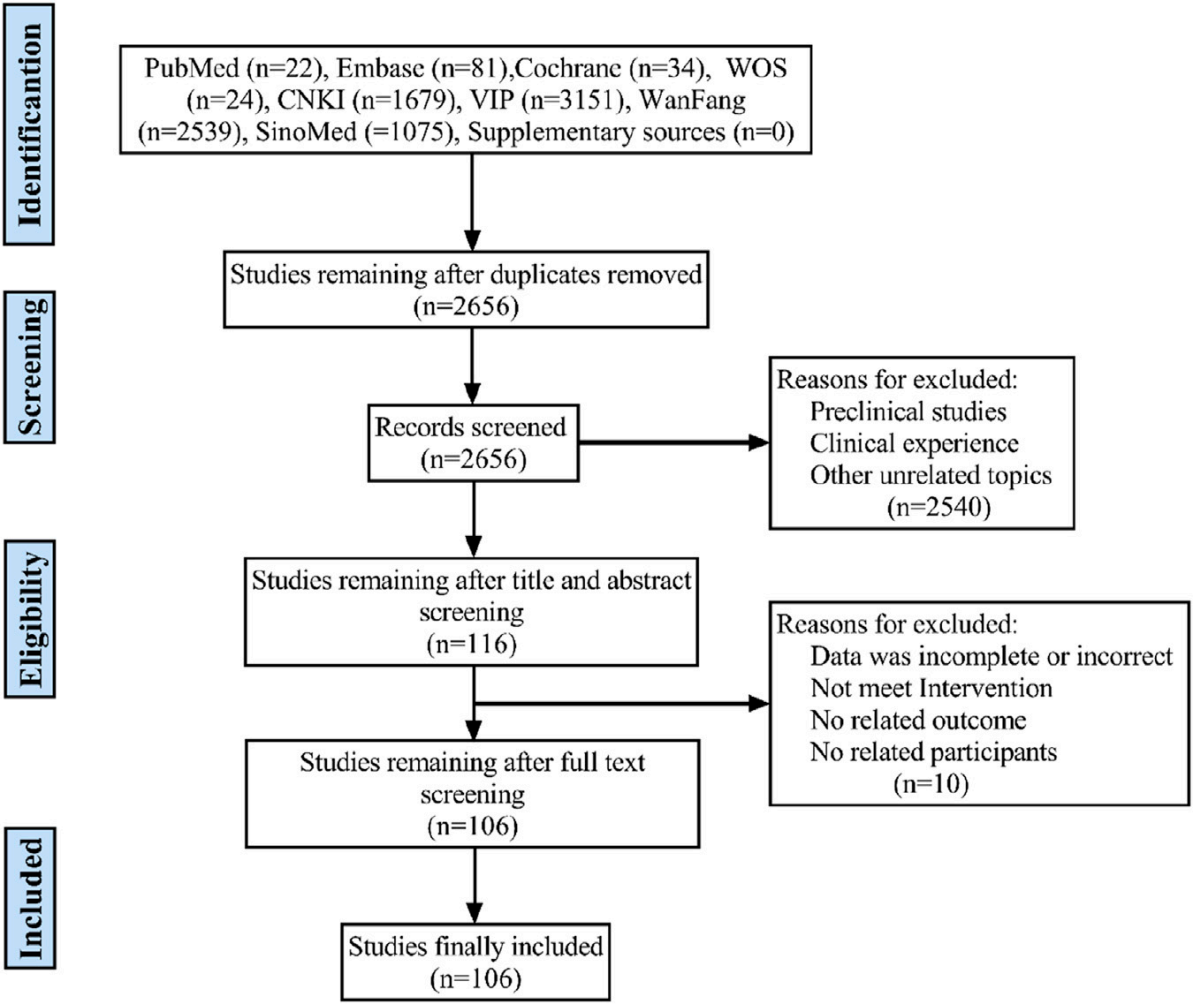
Flow diagram of eligible literature selection. CNKI, the China National Knowledge Infrastructure Database; WanFang, Wanfang Database; VIP, the Chinese Scientific Journals Full-text Database; SinoMed, the Chinese Biomedical Literature Database; n, number of publications.

A total of 8,605 articles were retrieved by searching eight databases. After removing duplicate articles, 2,656 articles remained for abstract screening. Excluding reviews, meta-analyses, systematic reviews, animal studies, and other u—elated topics, 116 articles were left for full-text review. Ultimately, 106 RCTs met the criteria for inclusion in this network meta-analysis. The detailed process of article selection is illustrated in Figure 1.

### 3.2 Study characteristic

A total of 106 RCTs were included in this research, involving 10,931 patients, with 5,640 in the experimental group and 5,291 in the control group. All included studies were conducted in China. The research covered 10 types of DSCIs, including DSDFSY + WM vs. WM (n = 44), DH + WM vs. WM (n = 31), DSCXQ + WM vs. WM (n = 9), STS + WM vs. WM (n = 8), DS + WM vs. WM (n = 2), SXPTT + WM vs. WM (n = 2), GXN + WM vs. WM (n = 4), XD + WM vs. WM (n = 1), DSFZ + WM vs. WM (n = 1), DH + WM vs. DS + WM (n = 1), DH + WM vs. FFDS + WM (n = 1), FFDS + WM vs. DH + WM (n = 1), and DSDFSY + WM vs. DS + WM (n = 1). The control group treatment was WM, mainly consisting of antiplatelet aggregation drugs, β-blockers, statins, nitrates, etc. Detailed information on the characteristics of the included studies is shown in Table 1.

**TABLE 1:** Characteristics of included studies.

Study ID	Sample size		Sex (M/F]		Average age		Therapy of experiment group	Menstruum	Therapy of control group	Course (days)		Outcomes	
	E	C	E	C	E	C							
Guo et al. (2016)	50	50	32/18	36/14	63.2 ± 2.1	62.9 ± 1.8	DSDFSY 200 mg + WM	5%GS/0.9%NS 250 mL	WM	14d		②	
Lv (2018)	30	30	20/10	22/8	72.25 ± 8.31	71.29 ± 8.28	DSDFSY 200 mg + WM	5%GS/0.9%NS 250 mL	WM	21d		①②⑤	
Wu et al. (2010)	39	33	24/15	19/14	69.5 ± 8.50		DSDFSY 200 mg + WM	5%GS/0.9%NS 250 mL	WM	14d		③	
Zhang et al. (2018)	170	172	88/82	92/80	58.60 ± 10.1	57.90 ± 9.4	DH 40 mL + WM	0.9% NS 250 mL	WM	15d		①③④	
Qiu et al. (2017)	40	40	42/38		61.6 ± 11.30		DSDFSY 200 mg + WM	5%GS/0.9%NS 250 mL	WM	14d		②③	
Xi et al. (2015)	35	35	21/14	17/18	55.3 ± 8.7	57.2 ± 10.3	SXPTT 100 mL + WM	—	WM	21d		②	
Bi et al. (2011)	62	62	80/44		68.3 ± 7.90		SXPTT 200 mL + WM	—	WM	21d		②	
Song et al. (2015)	40	40	20/20	22/18	54.11 ± 5.24	53.42 ± 5.68	DSCXQ 10 mL + WM	0.9% NS 250 mL	WM	14d		①②④	
Liu et al. (2015)	62	62	34/28	32/28	67.7 ± 4.3	68.8 ± 5.1	DSCXQ 10 mL + WM	0.9% NS 250 mL	WM	28d		①②	
Cai et al. (2014)	54	52	30/24	29/23	77 ± 6.8	76 ± 7.2	DSCXQ 10 mL + WM	0.9% NS 250 mL	WM	15d		①②	
Li and Yin (2012)	60	60	69/51		74 ± 7		DSCXQ 10 mL + WM	0.9% NS 250 mL	WM	6d		②	
Yu and Fang (2016)	47	47	26/21	27/20	—		DSCXQ 5–10 mL + WM	0.9% NS 250–500 mL	WM	20d		①②	
Ji (2013)	40	40	48/32		42.3 ± 6.7		DSCXQ 10 mL + WM	0.9% NS 250 mL	WM	10d		①②	
Liu and Xiao (2014)	83	77	48/35	45/32	65.3 ± 12.1	63.8 ± 11.3	DSDFSY 200 mg + WM	5%GS/0.9%NS 250 mL	WM	28d		①②	
Chen and Huang (2016a)	25	25	14/11	15/10	61.5 ± 5.7	62.3 ± 6.0	DSDFSY 200 mg + WM	5%GS/0.9%NS 250 mL	WM	14d		③④	
Wang and Bi (2017)	61	61	35/26	34/27	59.1 ± 6.6	58.4 ± 6.4	DSDFSY 200 mg + WM	5%GS/0.9%NS 250–500 mL	WM	14d		①③④	
Mao et al. (2016)	48	48	32/16	31/17	58.3 ± 7.5	57.4 ± 7.3	DSDFSY 100 mg + WM	5%GS/0.9%NS 250 mL	WM+0.9%NS250 mL	14d		①②	
Jiang et al. (2012)	30	30	—		—		DSDFSY 200 mg + WM	5%GS/0.9%NS 250 mL	WM	10d		①②	
Tao et al. (2024)	53	53	39/14	35/18	68.8	67.6	DSDFSY 100 mg + WM	0.9% NS 250 mL	WM	14d		①②	
Zhang et al. (2015a)	42	42	48/36		63.1 ± 8.8	65.4 ± 10.2	DSDFSY 100 mg + WM	0.9% NS 250 mL	WM	14d		①②④	
Li et al. (2018a)	76	76	49/27	52/24	58.1 ± 7.8	59.3 ± 8.2	DSDFSY 100 mg + WM	0.9% NS 250 mL	WM	1m		①②③	
Zhu (2019)	38	38	21/17	20/18	60.37 ± 8.89	59.87 ± 9.87	DSDFSY 150 mg + WM	5% GS 250 mL	WM	15d		②	
Liu et al. (2023)	51	51	30/21	28/23	62.98 ± 4.57	63.56 ± 4.89	DSDFSY 200 mg + WM	0.9% NS 250 mL	WM	7d		①②④	
Jiang (2022)	52	52	30/22	27/25	62.39 ± 6.43	62.45 ± 6.10	DSDFSY 200 mg + WM	5% GS 250 mL	WM	14d		①③④	
Jing (2014)	42	40	31/11	30/10	64.67 ± 7.45	64.33 ± 6.99	DSDFSY 200 mg + WM	5%GS/0.9%NS 250 mL	WM	14d		①②	
Xia and Zhang (2022)	43	43	23/20	22/21	71.52 ± 1.25	71.59 ± 2.13	DSDFSY 200 mg + WM	5%GS/0.9%NS 250 mL	WM	14d		①②④	
Jiao and Wang (2019)	40	40	24/16	23/17	49.57 ± 4.39	49.63 ± 4.57	DSDFSY 200 mg + WM	0.9% NS 250 mL	WM	14d		①④	
Pan et al. (2024)	66	66	39/27	40/26	61.3 ± 10.8	60.7 ± 10.4	DSDFSY 200 mg + WM	0.9% NS 200 mL	WM	14d		④	
Qiu (2020)	31	35	42/24		61.5 ± 11.4	62.1 ± 12.6	DSDFSY 200 mg + WM	0.9% NS 250 mL	WM	14d		①②④	
Xie et al. (2020)	53	53	29/24	27/26	57.42 ± 6.45	58.02 ± 6.49	DSDFSY 200 mg + WM	5% GS 250 mL	WM	28d		②④	
Yue et al. (2023)	51	51	26/25	28/23	61.53 ± 2.92	61.48 ± 2.81	DSDFSY 200 mg + WM	0.9% NS 250 mL	WM	28d		①④	
Chen et al. (2012)	40	40	47/33		53.2 ± 6.8		STS 12 mL + WM	0.9% NS 100 mL	WM	14d		②	
Yang and Ren (2010)	32	32	18/14	17/15	59 ± 11	60 ± 10	STS 12 mL + WM	5%GS/0.9%NS 250 mL	WM	7d		①②④	
Li et al. (2007)	37	36	39/34		67.4 ± 6.5		DS 20 mL + WM	—	WM	14d		②	
Li et al. (2009a)	92	90	55/37	54/36	61 ± 14	63 ± 11	DH 20 mL + WM	0.9% NS 250 mL	WM	14d		①②	
Huang (2017)	20	20	11/9	8/12	53.7 ± 6.5	54.3 ± 6.1	DH 20 mL + WM	0.9% NS 250 mL	WM	28d		②	
Xie and Cao (2011)	32	32	20/12	18/14	62.8 ± 8.2	63.0 ± 8.5	DH 30 mL + WM	0.9% NS 250 mL	WM	14d		①②	
Mei et al. (2006)	22	20	12/10	11/9	61.1 ± 9.5	61.6 ± 9.9	DH 40 mL + WM	5% GS 500 mL	WM	14d		②	
Liao et al. (2008)	40	30	19/21	17/13	65.1 ± 13.5	64.2 ± 10.3	DH 20 mL + WM	5%GS/0.9%NS 250 mL	WM	10d		②	
Zhao et al. (2015)	54	54	27/27	26/28	61.93 ± 4.41	62.53 ± 4.73	DH 30 mL + WM	0.9% NS 250 mL	WM	14d		①②⑤	
Zhang (2011)	60	60	32/28	31/29	61.21 ± 5.02	61.51 ± 7.86	DH 40 mL + WM	5%GS/0.9%NS 250 mL	WM	14d		①②④	
Yang and Shi (2009)	60	60	46/14	47/13	62.9 ± 6.9	63.1 ± 7.2	DH 30 mL + WM	5%GS/0.9%NS 250 mL	WM	14d		②④	
Peng et al. (2015)	45	45	26/19	27/18	63.2 ± 9.2	63.5 ± 9.0	DH 30 mL + WM	0.9% NS 250 mL	WM	14d		①②	
Fu and Jiang (2015)	49	47	27/22	26/21	46.17 ± 5.43	45.81 ± 4.79	DH 30 mL + WM	0.9% NS 250 mL	WM	10d		①②	
Wang and Wang (2020)	55	55	33/22	32/23	64.9 ± 7.1	65.3 ± 6.9	DH 20 mL + WM	0.9% NS 250 mL	WM	14d		①④	
Li et al. (2008)	80	50	48/32	28/22	60.3 ± 10.10	62.18 ± 10.50	DH 20 mL + WM	0.9% NS 250 mL	WM	14d		①②	
Zhao et al. (2011)	36	36	42/30		50.9 ± 8.60	60.2 ± 9.20	DH 40 mL + WM	5% GS 250 mL	WM	14d		①②	
Chen et al. (2015a)	65	65	43/22	49/16	68.00 ± 12.00	69.00 ± 14.00	DH 30 mL + WM	5%GS/0.9%NS 100 mL	WM	14d		①②	
Liu et al. (2012)	48	48	27/21	25/23	62.50 ± 3.60	62.9 ± 3.10	DH 40 mL + WM	5%GS/0.9%NS 250 mL	WM	15d		①②	
Li and Ge (2011)	44	42	50/36		64.5 ± 8		DH 20 mL + WM	10%GS/0.9%NS 250 mL	WM	10d		①②	
SHI and Liu (2012)	80	76	40/40	39/37	66–80	65–78	DH 40 mL + WM	0.9% NS 250 mL	WM	14d		①②	
Chen et al. (2015b)	94	73	59/35	47/26	64.83 ± 8.45	64.54 ± 9.64	DH 30 mL + WM	5%GS/0.9%NS 250 mL	WM	14d		①②④	
Xu et al. (2014)	46	46	30/16	32/14	53.40 ± 8.20	52.20 ± 9.60	GXN 20 mL + WM	5% GS 250 mL	WM	15d		①②	
Fu and Meng (2011)	25	22	13/12	11/11	—		GXN 20 mL + WM	5% GS 250 mL	WM	10d		①②	
Zhang et al. (2012)	44	44	22/22	26/18	22–41	20–44	DH 20 mL + WM	0.9% NS 250 mL	WM	10d		①	
Zhang (2016)	35	35	17/18	18/17	53.70 ± 4.30	54.10 ± 4.50	DSDFSY 100 mg + WM	0.9% NS 250 mL	WM	28d		①②④	
Hong et al. (2004)	90	30	52/38	17/13	62.95 ± 9.36	66.00 ± 8.96	XD 20 mL + WM	5%GS/0.9%NS 250 mL	WM	14d		①	
Lang (2012)	60	60	38/22	35/25	60.60 ± 8.20	62.50 ± 8.00	DSCXQ 20 mL + WM	—	WM	14d		①②④	
Yu and Jiao (2014)	62	62	41/21	40/22	50.30 ± 8.80	51.40 ± 7.90	DSDFSY 200 mg + WM	0.9% NS 250 mL	WM	14d		①②	
Lu and Ma (2023)	60	60	38/22	35/25	69.53 ± 5.18	69.34 ± 5.23	DSDFSY 200 mg + WM	5% GS 250 mL	WM	14d		①④	
Feng and Ji (2010)	20	20	31/9		55.18 ± 4.35		DSDFSY 100 mg + WM	5% GS 250 mL	WM	14d		①⑤⑥⑦	
Mei et al. (2014)	39	39	22/17	23/16	65.40 ± 4.20	66.20 ± 4.10	DSCXQ 10 mL + WM	5% GS 250 mL	WM	14d		②⑤	
Xu and Hao (2022)	30	30	20/10	17/13	65.73 ± 4.88	65.75 ± 4.76	DSCXQ 10 mL + WM	0.9% NS 250 mL	WM	28d		⑥	
Chen and Huang (2016b)	30	30	18/12	17/13	62.80 ± 6.50	63.10 ± 5.80	DSDFSY 200 mg + WM	5%GS/0.9%NS 250 mL	WM	14d		⑤	
Zhang (2010)	30	20	25/5	14/6	70.56 ± 2.25	71.21 ± 5.10	DSDFSY 200 mg + WM	5% GS 250 mL	WM	14d		⑤	
Wu et al. (2012)	30	30	19/11	17/13	59.91 ± 10.86	59.92 ± 9.03	DSDFSY 200 mg + WM	5%GS/0.9%NS 250 mL	WM	10d		①⑤	
Duan et al. (2010)	28	32	18/14	15/13	62.3	61.5	DSDFSY 200 mg + WM	5%GS/0.9%NS 250 mL	WM	10d		①⑥⑦	
Guan et al. (2019)	40	40	24/16	23/17	58.30 ± 7.30	57.90 ± 7.30	DSDFSY 200 mg + WM	5%GS/0.9%NS 250–500 mL	WM	14-28d		⑤⑥⑦	
Lin et al. (2020)	93	92	52/41	50/42	62.10 ± 7.20	62.40 ± 7.30	DSDFSY 200 mg + WM	5% GS 250 mL	WM	28d		①⑤	
Tan et al. (2009)	42	40	35/7	31/9	71.56 ± 8.25	70.18 ± 8.26	DSDFSY 200 mg + WM	5% GS 250 mL	WM	14d		⑤	
Pang et al. (2014)	42	42	26/16	25/17	73.40 ± 7.30	73.80 ± 7.50	DSDFSY 200 mg + WM	5% GS 250 mL	WM	14d		①⑤	
Liu (2017)	40	40	23/17	22/18	59.12 ± 2.13	60.29 ± 2.24	DSDFSY 200 mg + WM	5%GS/0.9%NS 250 mL	WM	14d		①⑤	
Ye et al. (2021)	74	76	43/31	46/30	59.64 ± 6.98	60.17 ± 7.05	DSDFSY 200 mg + WM	0.9% NS 250 mL	WM	10d		⑥	
Lun et al. (2011)	30	30	19/11	18/12	60.11 ± 11.00	62.31 ± 8.30	DSDFSY 200 mg + WM	5%GS/0.9%NS 250 mL	WM	14d		①⑤	
Qiu et al. (2018)	55	55	30/25	31/24	58.93 ± 7.51	59.31 ± 7.29	DSDFSY 200 mg + WM	0.9% NS 250 mL	WM	56d		⑥⑦	
Chen et al. (2020)	60	60	33/27	34/26	62.86 ± 4.15	62.55 ± 4.23	DSDFSY 200 mg + WM	5%GS/0.9%NS 250 mL	WM	14d		①	
Song et al. (2017)	42	31	35/38		63.01 ± 9.25	63.75 ± 11.83	DSDFSY 200 mg + WM	5%GS/0.9%NS 250 mL	WM	14d		①④⑤	
Yang et al. (2010)	22	20	12/10	12/8	71.56 ± 8.20	70.18 ± 8.26	DSDFSY 100 mg + WM	5% GS 250 mL	WM	14d		①⑤	
Zhu and He (2002)	42	40	24/18	24/16	54.00 ± 8.00	55.00 ± 8.00	DSFZ 400 mg + WM	5% GS 500 mL	WM	15d		①⑥	
Jin et al. (2012)	50	50	62/48		66 ± 8.5		STS 20 mL + WM	5%GS 250 mL	WM	14d		⑤	
Wei and Shen (2014)	60	60	38/22	36/24	49.18 ± 12.50	52.36 ± 10.18	STS 12 mL + WM	—	WM	14d		①⑥⑦	
Fang et al. (2018)	33	33	17/16	16/17	58.60 ± 4.80	57.90 ± 5.40	STS 16 mL + WM	0.9% NS 250 mL	WM	14d		⑤	
Mou (2011)	34	37	41/30		53.57 ± 12.83		STS 12 mL + WM	5% GS 250 mL	WM	14d		①	
Qi et al. (2001)	80	80	42/38		65.59 ± 7.39		DS 10 mL + WM	0.9% NS 100 mL	WM	10d		⑥⑦	
Hu and Liu (2011)	36	36	49/23		57.6 ± 4.5		DH 30 mL + WM	0.9% NS 100 mL	WM	14d		②⑤	
Luo and Huang (2011)	39	39	48/30		67.8		DH 20 mL + WM	0.9% NS 100 mL	WM	14d		①⑤	
Li and Yang (2008)	100	100	52/48	53/47	62.56 ± 6.53	62.53 ± 5.42	DH 40 mL + WM	5% GS 250 mL	WM	14d		①⑤	
Su et al. (2011)	50	48	45/5	43/5	73.46 ± 9.34	72.56 ± 9.62	DH 30 mL + WM	—	WM	28d		⑥⑦	
Li et al. (2009b)	67	67	34/33	38/29	55.20 ± 4.70	54.80 ± 5.20	GXN 20 mL + WM	5%GS/0.9%NS 250 mL	WM	10d		①⑥⑦	
Li and Wang (2007)	58	62	31/27	33/29	66.89 ± 10.79	65.79 ± 9.98	GXN 20 mL + WM	—	WM	10d		⑥⑦	
Chen et al. (2009)	44	38	—		—		DSDFSY 200 mg + WM	5%GS/0.9%NS 250 mL	WM	14d		①	
Liu et al. (2021)	364	182	407/206	188/117	59.60 ± 6.93	60.01 ± 6.75	DH 40 mL + WM	0.9% NS 250 mL	WM+0.9% NS 250 mL	14d		②	
Li et al. (2018b)	80	80	53/27	55/25	56.80 ± 11.40	58.9 ± 12.30	DH 30 mL + WM	5%GS/0.9%NS 250 mL	WM	14d		①	
Sun and Zhang (2024)	52	57	32/21	32/25	55.20 ± 7.60	56.20 ± 8.70	DH	—	WM	14d		①	
Sun et al. (2014)	36	36	—		—		DH 20 mL + WM	5%GS/0.9%NS 100 mL	WM+5%GS/0.9%NS 100 mL	14d		①	
Li et al. (2014)	36	36	—		—		STS 16 mL + WM	—	WM	14d		②	
Yao (2022)	40	40	45/72	40/73	—		DSDFSY 200 mg + WM	0.9% NS 200 mL	WM	0.5m		④⑤	
Gao et al. (2009)	42	40	—		—		DH 20 mL + WM	0.9% NS 200 mL	WM	14d		②	
Chen et al. (2021)	80	80	52/28	46/34	70.06 ± 8.15	70.25 ± 8.17	DSDFSY 200 mg + WM	5%GS/0.9%NS 250 mL	WM	14d		①②④	
Zhang (2017)	50	50	30/20	28/22	67.5 ± 5.40	66.2 ± 6.19	STS 12 mL + WM	0.9% NS 250 mL	WM	14d		②	
Wang (2009)	50	50	27/23	29/21	57.2 ± 8.7	56.8 ± 8.7	DH 20 mL + WM	5% GS 200 mL	WM	14d		②	
Liu and Wang (2013)	40	40	23/17	25/15	47.3	48.8	DH 30 mL + WM	5%GS/0.9%NS 250 mL	WM	10d		①②	
Cao and Liu (2013)	43	43	22/21	19/24	—		DH 30 mL + WM	5%GS/0.9%NS 250 mL	WM + DS 30 mL	14d		②⑤	
Yan and Zeng (2009)	30	30	42/18		58.1 ± 10.6		DH 20 mL + WM	5%GS/0.9%NS 500 mL	WM + FFDS 20 mL	28d		①⑤⑥⑦	
Zhang et al. (2010)	40	40	—		—		FFDS 20 mL + WM	5% GS 250 mL	WM + DH 30 mL	14d		①②	
Zhou and Guo (2018)	96	96	54/42	58/38	54.7 ± 11.6	55.4 ± 10.8	DSDFSY 200 mg + WM	0.9% NS 250 mL	WM + DS 30 mL	14d		①⑤	

M, male; F, female; E, experimental group; C, control; ① clinical effectiveness rate; ②high-sensitivity C-reactive protein (hs-CRP); ③interleukin-1 (IL-1); ④interleukin-6 (IL-6); ⑤nitric oxide (NO); ⑥superoxide dismutase (SOD); ⑦malondialdehyde (MDA).

### 3.3 Quality evaluation

**FIGURE 2:**
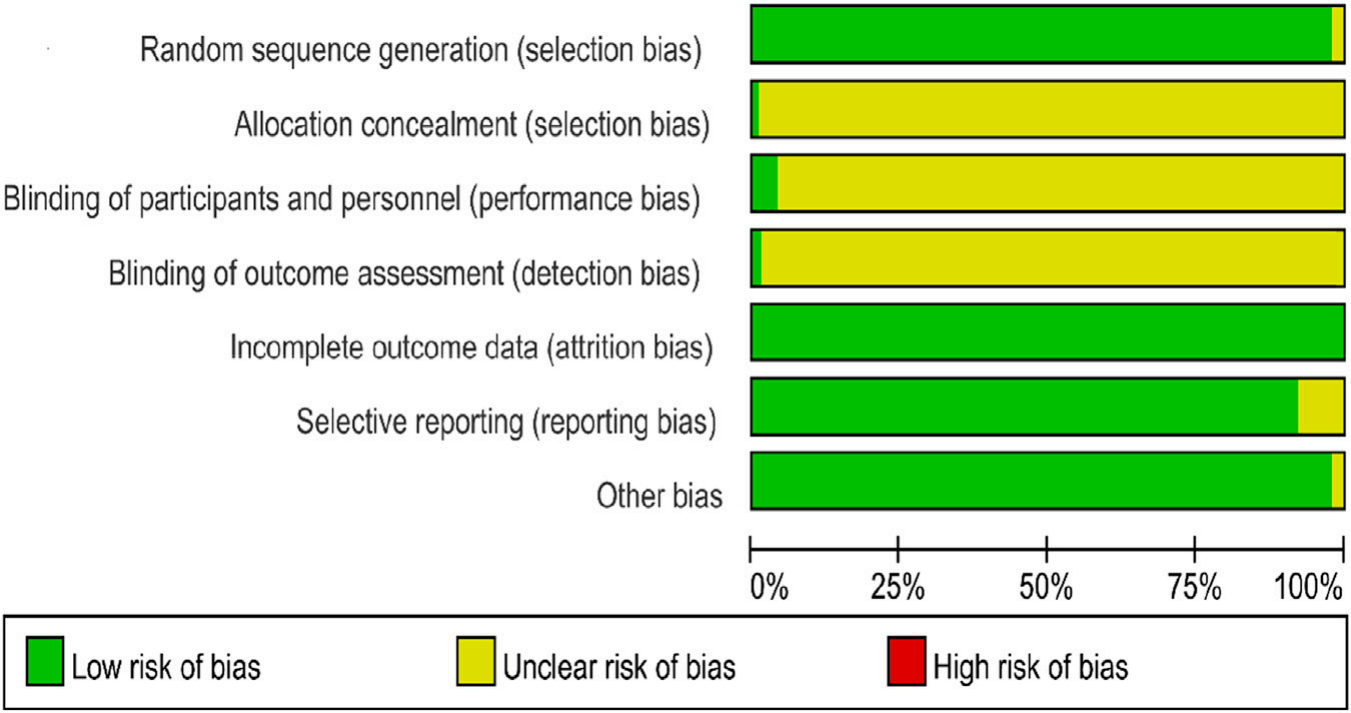
Results of risk of bias evaluation of included studies.

A risk of bias assessment was conducted on the 106 included studies. (1) Selection bias: Two studies did not mention randomization and were considered “unclear risk,” while the remaining studies mentioned random allocation but may not have reported the specific randomization method; these were also evaluated as “low risk”. (2) Allocation concealment: One study mentioned using allocation concealment and was considered “low risk”, while the rest did not mention this information and were deemed “unclear risk”. (3) Performance bias: Four studies explicitly stated that the trial design was “single-blind”, thus considered “low risk”. Studies that did not report relevant information were deemed “unclear risk”. (4) Detection bias: One study mentioned blinding the outcome assessors, considered “low risk”, while other studies did not mention this information and were deemed “unclear risk”. (5) Attrition bias: All included studies had no incomplete data, so the risk of attrition bias was considered “low risk”. (6) Reporting bias: Eight studies reported fewer outcomes, potentially indicating reporting bias, and were evaluated as “unclear risk”, while the remaining studies were “low risk.” (7) Other biases: Two studies did not report whether the experimental and control groups were comparable at baseline and were evaluated as “unclear risk”, while the remaining studies were considered “low risk”. Overall, the quality of the included studies was suboptimal, with summary results shown in Figure 2 and ROB 2.0 evaluation results in Supplementary table.

### 3.4 Results of network meta-analysis

#### 3.4.1 Consistency testing

None of the interventions in this study formed a closed loop, so consistency testing was not required.

#### 3.4.2 Clinical effectiveness rate

**FIGURE 3:**
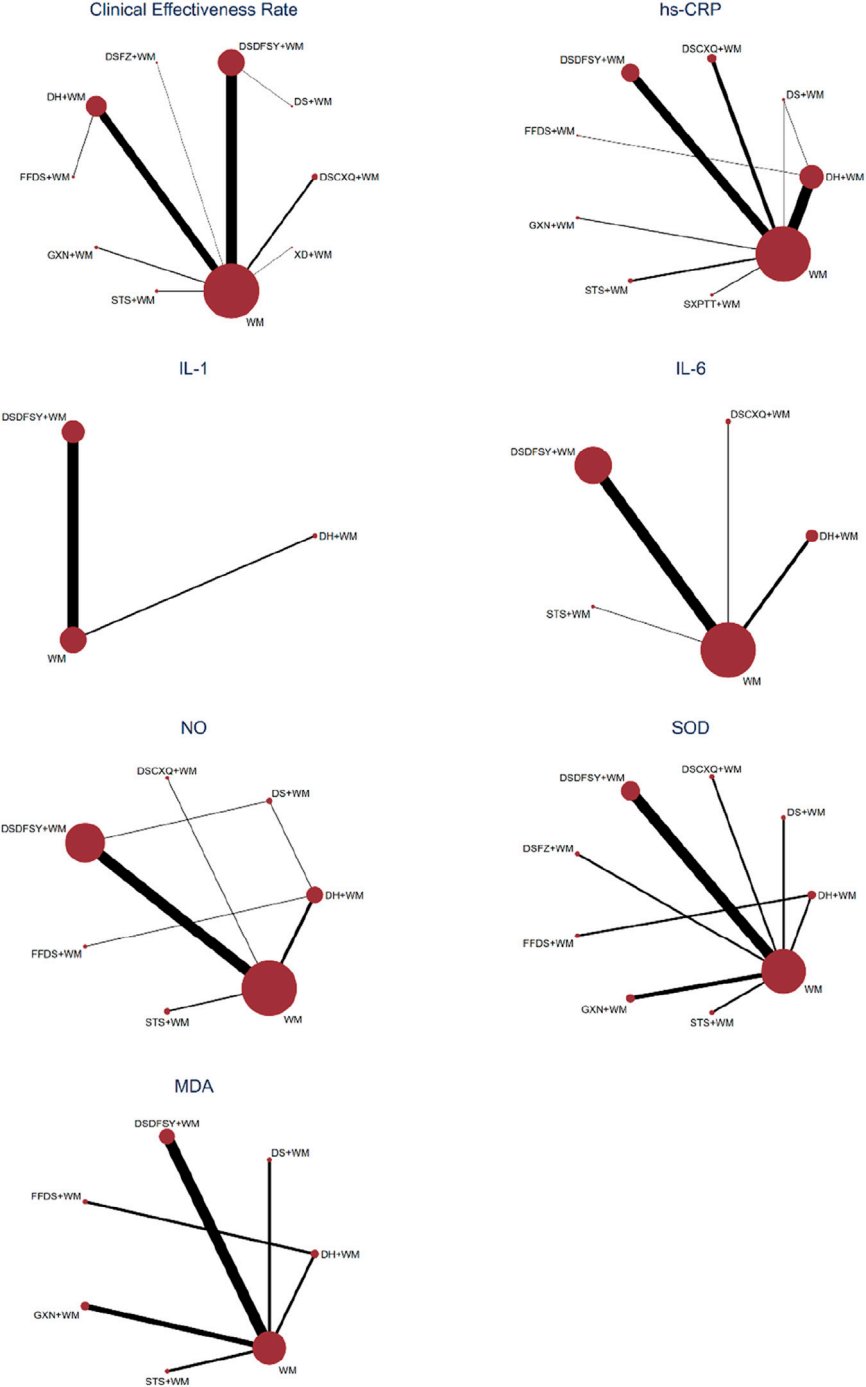
Network diagram of various outcomes. WM, western medicine; DH, Danhong injection; DS, Danshen injection; DSCXQ, Danshenchuanxiongqin injection; DSDFSY, Dansenduofensuanyan injection; DSFZ, Danshenfen injection; FFDS, Fufang Danshen injection; GXN, Guanxinning injection; STS, Sodium Tanshinone IIA Sulfonate injection; XD, Xiangdan injection; SXPTT, Shenxiongputaotang injection. hs-CRP, high-sensitivity C-reactive protein; IL-1, interleukin-1; IL-6, interleukin-6; NO, nitric oxide; SOD, superoxide dismutase; MDA, malondialdehyde.

**FIGURE 4:**
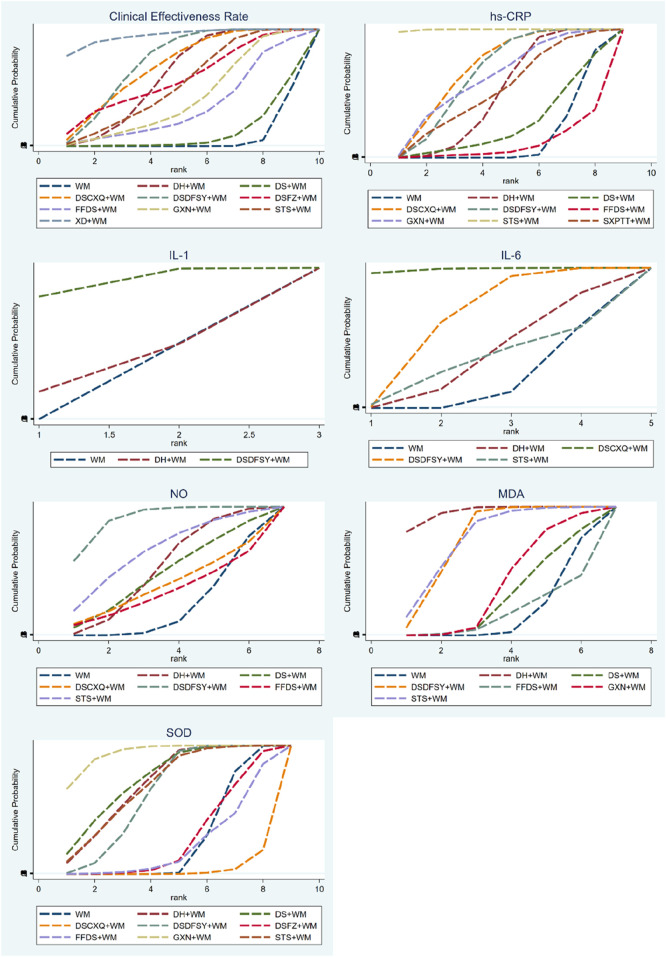
SUCRA for different outcomes.

A total of 69 randomized controlled trials involving 9 types of DSCIs were included in the analysis of clinical effectiveness rate: DSDFSY + WM vs. WM (n = 30), DSFZ + WM vs. WM (n = 1), DH + WM vs. FFDS + WM (n = 1), FFDS + WM vs. DH + WM (n = 1), DH + WM vs. WM (n = 22), XD + WM vs. WM (n = 1), DSDFSY + WM vs. DS + WM (n = 1), DSCXQ + WM vs. WM (n = 6), GXN + WM vs. WM (n = 3), and STS + WM vs. WM (n = 3). The network relationship diagram is shown in Figure 3. Connections between nodes represent direct comparative evidence between the two interventions, while the absence of a connection indicates no direct comparison. The thickness of the lines indicates the number of included studies comparing each treatment, and the size of the circles represents the sample size of the population using each intervention. Except for the DS + WM and FFDS + WM groups, all other DSCIs combined with WM showed superior clinical efficacy compared to WM alone. Additionally, compared to DS + WM, the clinical efficacy rates of DSCXQ + WM, DSDFSY + WM, XD + WM, and DH + WM were significantly higher. No significant differences were observed between other interventions (Table 2). According to the SUCRA probabilities ranking, XD + WM (94.0%) was the most likely to be the best intervention to improve the clinical efficacy rate, followed by DSDFSY + WM (73.1%) > DSCXQ + WM (68.7%) > DH + WM (60.7%) > DSFZ + WM (58.4%) > STS + WM (53.7%) > GXN + WM (40.5%) > FFDS + WM (33.7%) > DS + WM (10.9%) > WM (6.2%). The specific results are shown in the Table 3 and Figure 4.

**TABLE 2:** OR/MD (95%CIs) of all interventions. The bolded and underlined results indicate statistical significance.

Comparison		Clinical effectiveness rate	hs-CRP	IL-1	IL-6	NO	SOD	MDA
DH + WM	vs. WM	**3.82 (2.97, 4.92)**	**−1.42 (-1.98, -0.86)**	0.16 (−141.93, 141.61)	−6.76 (−28.17, 14.66)	9.74 (−5.90, 25.38)	**10.28 (2.17, 18.40)**	**−2.40 (-3.52, -1.28)**
DS + WM		1.11 (0.41, 2.98)	−0.29 (−2.49, 1.91)			6.64 (−17.67, 30.95)	**11.49 (2.31, 20.67)**	−0.25 (−1.29, 0.79)
DSCXQ + WM		**4.27 (2.48, 7.35)**	**−1.94 (-2.83,-1.05)**		**−62.11 (-99.46, -24.77)**	4.55 (−28.67, 37.77)	**−9.39 (-18.24,-0.54)**	
DSDFSY + WM		**4.36 (3.43, 5.56)**	**−1.82 (-2.42, -1.21)**	**−67.20 (-125.44,-8.96)**	**−18.73 (-31.38, -6.08)**	**25.76 (17.05, 34.47)**	**8.92 (4.86, 12.99)**	**−1.52 (-2.11,-0.93)**
DSFZ + WM		**3.60 (1.04, 12.48)**					0.16 (−7.79, 8.11)	
FFDS + WM		2.11 (0.66, 6.73)	0.75 (−1.84, 3.34)			1.23 (−35.51, 37.96)	−1.69 (−13.80, 10.43)	0.18 (−1.37, 1.73)
GXN + WM		**2.63 (1.15, 5.97)**	−1.81 (−3.66, 0.03)				**16.61 (10.15 23.08)**	−0.45 (−1.21, 0.32)
STS + WM		**3.38 (1.82, 6.30)**	**−4.66 (-6.41, -2.92)**		−3.62 (−50.83, 43.59)	16.72 (−6.99, 40.42)	**10.11 (1.04, 19.18)**	**−1.58 (-2.77, -0.39)**
XD + WM		**8.97 (3.23, 24.89)**						
SXPTT + WM			−1.45 (−3.26, 0.36)					
DS + WM	vs. DH + WM	**0.29 (0.10, 0.80)**	1.13 (−1.05, 3.32)			−3.10 (−27.64, 21.43)	1.21 (−11.05, 13.46)	**2.15 (0.62, 3.67)**
DSCXQ + WM		1.12 (0.61, 2.03)	−0.52 (−1.57, 0.53)		**−55.36 (-98.36, -12.35)**	−5.19 (−41.91, 31.53)	**−19.67 (-31.68, -7.66)**	
DSDFSY + WM		1.14 (0.80, 1.62)	−0.39 (−1.22, 0.43)	−67.04 (-220.31, 86.23)	−11.97 (-36.83, 12.88)	16.02 (−1.50, 33.54)	−1.36 (−10.44, 7.72)	0.88 (−0.38, 2.15)
DSFZ + WM		0.94 (0.27, 3.35)					−10.12 (−21.49, 1.24)	
FFDS + WM		0.55 (0.18, 1.71)	2.17 (−0.36, 4.70)			−8.52 (−41.79, 24.76)	**−11.97 (-20.97, -2.97)**	**2.58 (1.50, 3.66)**
GXN + WM		0.69 (0.29, 1.62)	−0.39 (−2.32, 1.54)				6.33 (−4.05, 16.71)	**1.95 (0.60, 3.30)**
STS + WM		0.88 (0.45, 1.73)	**−3.24 (-5.07, -1.41)**		3.14 (−48.70, 54.98)	6.98 (−21.42, 35.38)	−0.17 (−12.34, 12.00)	0.82 (−0.81, 2.45)
XD + WM		2.35 (0.82, 6.71)						
SXPTT + WM			−0.02 (−1.92, 1.87)					
DSCXQ + WM	vs. DS + WM	**3.86 (1.25, 11.97)**	−1.65 (-4.02, 0.72)			−2.09 (−43.26, 39.08)	**−20.88 (-33.64, -8.12)**	
DSDFSY + WM		**3.95 (1.51, 10.33)**	−1.52 (-3.80, 0.75)			19.12 (−5.32, 43.57)	−2.57 (−12.61, 7.48)	**−1.27 (-2.46, -0.07)**
DSFZ + WM		3.26 (0.66, 15.98)					−11.33 (−23.48, 0.82)	
FFDS + WM		1.91 (0.42, 8.78)	1.04 (−2.30, 4.38)			‘-5.41 (−46.74, 35.92)	−13.18 (−28.38, 2.03)	0.43 (−1.44, 2.30)
GXN + WM		2.37 (0.65, 8.61)	−1.52 (−4.39, 1.35)				5.12 (−6.11,16.35)	−0.20 (−1.49, 1.09)
STS + WM		3.06 (0.95, 9.86)	**−4.37 (-7.17, -1.58)**			10.08 (−23.87, 44.03)	−1.38 (−14.28, 11.52)	−1.33 (−2.91, 0.25)
XD + WM		**8.11 (1.95, 33.67)**						
SXPTT + WM			−1.16 (−4.00, 1.69)					
DSDFSY + WM	vs. DSCXQ + WM	1.02 (0.56, 1.85)	0.13 (−0.95, 1.20)		**43.38 (4.28, 82.49)**	21.21 (−13.14, 55.56)	**18.31 (8.57, 28.06)**	
DSFZ + WM		0.84 (0.22, 3.27)					9.55 (−2.35, 21.45)	
FFDS + WM		0.49 (0.14, 1.78)	2.69 (−0.05, 5.43)			−3.32 (−52.86, 46.21)	7.70 (−7.30, 22.71)	
GXN + WM		0.61 (0.23, 1.65)	0.13 (−1.92, 2.18)				**26.00 (15.04,36.97)**	
STS + WM		0.79 (0.35, 1.81)	**−2.72 (-4.67, -0.77)**		58.49 (−1.70, 118.69)	12.17 (−28.65, 52.98)	**19.50 (6.83, 32.17)**	
XD + WM		2.10 (0.66,6.68)						
SXPTT + WM			0.50 (−1.52, 2.51)					
DSFZ + WM	vs. DSDFSY + WM	0.83 (0.23, 2.93)					−8.76 (−17.70, 0.17)	
FFDS + WM		0.48 (0.15, 1.58)	2.56 (−0.10, 5.22)			−24.54 (−62.11, 13.04)	−10.61 (−23.39, 2.17)	**1.70 (0.04, 3.36)**
GXN + WM		0.60 (0.26, 1.42)	0.00 (−1.94, 1.94)				**7.69 (0.05, 15.33)**	**1.07 (0.10, 2.03)**
STS + WM		0.77 (0.40, 1.51)	**−2.85 (-4.69, -1.01)**		15.11 (−33.77, 63.99)	−9.04 (−34.30, 16.21)	1.19 (−8.75, 11.12)	−0.06 (−1.39, 1.26)
XD + WM		2.05 (0.72, 5.87)						
SXPTT + WM			0.37 (−1.54, 2.28)					
FFDS + WM	vs. DSFZ + WM	0.59 (0.11, 3.20)					−1.85 (−16.34, 12.65)	
GXN + WM		0.73 (0.16,3.23)					**16.45 (6.20, 26.70)**	
STS + WM		0.94 (0.23,3.76)					9.95 (−2.11,22.01)	
XD + WM		2.49 (0.50,12.42)						
SXPTT + WM								
GXN + WM	vs. FFDS + WM	1.24 (0.30, 5.14)	−2.56 (−5.74, 0.62)				**18.30 (4.57, 32.03)**	−0.63 (−2.36, 1.10)
STS + WM		1.60 (0.43, 5.96)	**−5.41 (-8.53, -2.29)**			15.49 (−28.23, 59.21)	11.80 (−3.34, 26.93)	−1.76 (−3.72, 0.20)
XD + WM		4.24 (0.91, 19.87)						
SXPTT + WM			−2.19 (−5.35, 0.97)					
STS + WM	vs. GXN + WM	1.29 (0.46, 3.61)	**−2.85 (-5.39, -0.31)**				−6.50 (−17.64, 4.63)	−1.13 (−2.54, 0.28)
XD + WM		3.42 (0.92, 12.67)						
SXPTT + WM			0.37 (−2.22, 2.95)					
XD + WM	vs. STS + WM	2.65 (0.80, 8.76)						
SXPTT + WM			3.22 (0.71, 5.73)					
SXPTT + WM	vs. XD + WM							

**TABLE 3:** SUCRA (%) of all therapeutic measures. The redder color in the table means the higher the ranking of the corresponding intervention.

	Clinical effectiveness rate	hs-CRP	IL-1	IL-6	NO	SOD	MDA
WM	6.2	14.9	25.5	17.8	21.5	26.3	17.4
DH + WM	60.7	49.9	34.2	36.7	52.1	70.4	95.8
DS + WM	10.9	25.4			47.7	74.1	30.1
DSCXQ + WM	68.7	69.6		98.9	38.8	3	
DSDFSY + WM	73.1	65.7	90.3	64.1	90.7	62.8	75.2
DSFZ + WM	58.4					27.8	
FFDS + WM	33.7	9.9			33.5	22.5	17
GXN + WM	40.5	62.4				94	39.2
STS + WM	53.7	99.6		32.5	65.7	69.1	75.3
XD + WM	94						
SXPTT + WM		52.7					

#### 3.4.3 Hs-CRP

A total of 59 randomized controlled trials involving 8 types of DSCIs were analyzed for hs-CRP: DH + WM vs. WM (n = 22), DS + WM vs. WM (n = 1), DSCXQ + WM vs. WM (n = 8), DSDFSY + WM vs. WM (n = 18), GXN + WM vs. WM (n = 2), STS + WM vs. WM (n = 4), SXPTT + WM vs. WM (n = 2), FFDS + WM vs. DH + WM (n = 1), and DH + WM vs. DS + WM (n = 1). The network relationship diagram is shown in Figure 3. DH, DSCXQ, DSDFSY, and STS combined with WM were superior to WM alone in reducing hs-CRP. Moreover, STS + WM was more effective than DH + WM, DS + WM, DSCXQ + WM, DSDFSY + WM, FFDS + WM, or GXN + WM in reducing hs-CRP, with statistically significant differences (Table 2). According to the SUCRA probability ranking, STS + WM (99.6%) is most likely to be the best intervention for reducing hs-CRP, followed by DSCXQ + WM (69.6%) > DSDFSY + WM (65.7%) > GXN + WM (62.4%) > SXPTT + WM (52.7%) > DH + WM (49.9%) > DS + WM (25.4%) > WM (14.9%) > FFDS + WM (9.9%). The specific results are shown in the Table 3 and Figure 4.

#### 3.4.4 IL-1

7 randomized controlled trials involving IL-1, including 2 types of DSCIs [DH + WM vs. WM (n = 1) and DSDFSY + WM vs. WM (n = 6)], were analyzed. The network relationship diagram is shown in Figure 3. DSDFSY + WM was more effective than WM alone in reducing IL-1, with statistically significant differences (Table 2). According to the SUCRA probability ranking, DSDFSY + WM (90.3%) is most likely to be the best intervention for reducing IL-1, followed by DH + WM (34.2%) and WM (25.5%). The specific results are shown in the Table 3 and Figure 4.

#### 3.4.5 IL-6

24 randomized controlled trials involving 4 types of DSCIs were analyzed for IL-6: DH + WM vs. WM (n = 5), DSCXQ + WM vs. WM (n = 2), DSDFSY + WM vs. WM (n = 16), and STS + WM vs. WM (n = 1). The network relationship diagram is shown in Figure 3. DSCXQ and DSDFSY combined with WM were more effective than WM alone in reducing IL-6. DSCXQ + WM was more effective than DH + WM in reducing IL-6, while DSDFSY + WM was less effective than DSCXQ + WM (Table 2). According to the SUCRA probability ranking, DSCXQ + WM (98.9%) is most likely to be the best intervention for reducing IL-6, followed by DSDFSY + WM (64.1%) > DH + WM (36.7%) > STS + WM (32.5%) > WM (17.8%). The specific results are shown in the Table 3 and Figure 4.

#### 3.4.6 NO

24 randomized controlled trials involving 6 types of DSCIs were analyzed for NO: DH + WM vs. WM (n = 4), DSCXQ + WM vs. WM (n = 1), DSDFSY + WM vs. WM (n = 14), STS + WM vs. WM (n = 2), DH + WM vs. FFDS + WM (n = 1), DH + WM vs. DS + WM (n = 1), and DSDFSY + WM vs. DS + WM (n = 1). The network relationship diagram is shown in Figure 3. DSDFSY + WM was more effective than WM alone in increasing NO (Table 2). According to the SUCRA probability ranking, DSDFSY + WM (90.7%) is most likely to be the best intervention for increasing NO, followed by STS + WM (65.7%) > DH + WM (52.1%) > DS + WM (47.7%) > DSCXQ + WM (38.8%) > FFDS + WM (33.5%) > WM (21.5%). The specific results are shown in the Table 3 and Figure 4.

#### 3.4.7 SOD

13 randomized controlled trials involving 8 types of DSCIs were analyzed for SOD: DH + WM vs. WM (n = 1), DS + WM vs. WM (n = 1), DSCXQ + WM vs. WM (n = 1), DSDFSY + WM vs. WM (n = 5), GXN + WM vs. WM (n = 2), STS + WM vs. WM (n = 1), DSFZ + WM vs. WM (n = 1), and FFDS + WM vs. DH + WM (n = 1). The network relationship diagram is shown in Figure 3. Except for DSFZ, FFDS, and DSCXQ, other types of DSCIs combined with WM were more effective than WM alone in increasing SOD (Table 2). According to the SUCRA probability ranking, GXN + WM (94.0%) is most likely to be the best intervention for increasing SOD, followed by DS + WM (74.1%) > DH + WM (70.4%) > STS + WM (69.1%) > DSDFSY + WM (62.8%) > DSFZ + WM (27.8%) > WM (26.3%) > FFDS + WM (22.5%) > DSCXQ + WM (3.00%). The specific results are shown in the Table 3 and Figure 4.

#### 3.4.8 MDA

10 randomized controlled trials involving 6 types of DSCIs were analyzed for MDA: DH + WM vs. WM (n = 1), DS + WM vs. WM (n = 1), DSDFSY + WM vs. WM (n = 4), GXN + WM vs. WM (n = 2), STS + WM vs. WM (n = 1), and FFDS + WM vs. DH + WM (n = 1). The network relationship diagram is shown in Figure 3. DH, DSDFSY, and STS combined with WM were more effective than WM alone in reducing MDA (Table 2). According to the SUCRA probability ranking, DH + WM (95.8%) is most likely to be the best intervention for reducing MDA, followed by STS + WM (75.3%) > DSDFSY + WM (75.2%) > GXN + WM (39.2%) > DSCXQ + WM (30.1%) > WM (17.4%) > FFDS + WM (17.0%). The specific results are shown in the Table 3 and Figure 4.

#### 3.4.9 Adverse reactions

A total of 49 studies reported adverse reaction events, with 17 studies detailing specific adverse reactions. These included the following manifestations: circulatory system: dizziness, headache, palpitations, fatigue; digestive system: nausea, abdominal distension, vomiting; peripheral vasculature: facial flushing; skin: rash, bruising. The specific results are shown in Table 4.

**TABLE 4:** Adverse reactions.

	Number	Dizziness	Headache	Palpitations	Fatigue	Nausea	Abdominal distension	Vomiting	Facial flushing	Rash	Bruising	Others
Zhang et al. (2018)	35					5					6	
Jiang et al. (2012)	48	1										
Li et al. (2018a)	76					1	1					4
Jiao and Wang (2019)	40					1						
Xie et al. (2020)	53		1		1							
Yue et al. (2023)	51	2	1			2						
Xie and Cao (2011)	32		1						1			
SHI and Liu (2012)	80	8	3	6								
Lu and Ma (2023)	60	1						2		2		
Duan et al. (2010)	28				1							
Guan et al. (2019)	40			1		1	1					
Fang et al. (2018)	33		1									
Mou (2011)	80	3			1	1						
Chen et al. (2009)	44			2					1			
Li et al. (2018b)	80	1							2			
Yao (2022)	40	1		1		1						
Zhou and Guo (2018)	96			2		2	4			4		
Summary		17	7	12	3	14	6	2	4	6	6	4

#### 3.4.10 Publication bias

**FIGURE 5:**
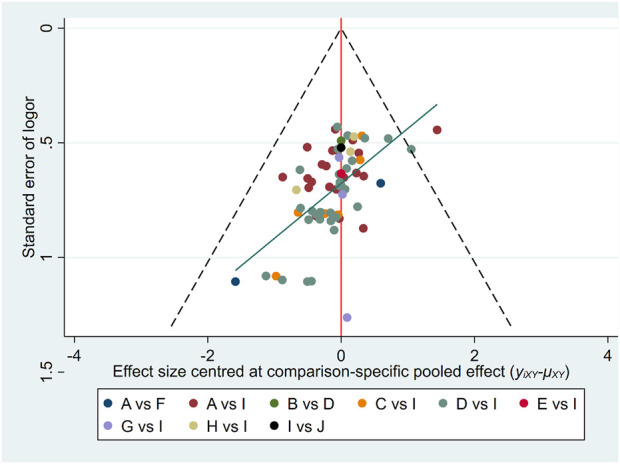
Funnel plots of clinical effectiveness rate. The vertical axis represents “standard error of effect size” and horizontal axis represents “effect size centred at comparison-specific pooled effect (y_ixy_-μ_xy_).” A, DH + WM; B, DS + WM; C, DSCXQ + WM; D, DSDFSY + WM; E, DSFZ + WM; F, FFDS + WM; G, GXN + WM; H, STS + WM; I, WM; J, XD + WM.

A funnel plot was used to test for publication bias in this study (Figure 5 and Supplementary Figure). As shown in the figure, most points are concentrated in the middle, with a few at the bottom, indicating that some studies had small sample sizes, which may lead to bias. Additionally, the points in the funnel plot are asymmetrically distributed relative to the centerline, and the regression line forms an angle with the centerline, suggesting the presence of publication bias in this study.

## 4 Discussion

Although DSCIs are widely used in China to treat CHD, they are mostly used as adjunctive therapies and rarely used alone. Their acceptance in other countries is relatively poor. The reasons for this may include: ① Although multiple clinical studies have confirmed the efficacy of DSCIs, the trial designs are not rigorous enough, and there is still a lack of large-scale, high-quality evidence-based medical evidence to verify their efficacy and safety. ② Most DSCIs are compound preparations containing various active metabolites, making their mechanisms of action complex. Notably, current basic research is often focused on simple target points and pathways, lacking in-depth studies on pharmacokinetics and pharmacological and toxicological mechanisms. ③ The complex composition of traditional Chinese medicine injections increases the probability of adverse reactions when used in combination with other drugs, limiting their clinical use by physicians. ④ There are many types of DSCIs, with both similarities and differences, making clinical drug selection challenging.

Based on these issues, this study employs the NMA method to fully utilize existing clinical research and compare the efficacy and safety of multiple DSCIs. By pooling small sample data, we expand the sample size, reduce bias and errors, improve statistical power, and obtain more reliable results. Additionally, the NMA results provide efficacy rankings for different outcome indicators, offering reference suggestions for clinical drug selection and helping clinicians understand the associations and differences between various treatment options.

This study used the NMA method to evaluate 106 randomized controlled trials that met the inclusion and exclusion criteria, involving 10 types of DSCIs. The outcomes included clinical efficacy, inflammatory markers (hs-CRP, IL-1, IL-6), oxidative stress markers (NO, SOD, MDA), and safety. The NMA results indicate that seven types of DSCIs combined with WM achieved better efficacy than WM alone. According to the SUCRA probability ranking, XD + WM showed the most significant improvement in clinical efficacy, followed by DSDFSY + WM and DSCXQ + WM. However, the relatively small sample size for XD + WM might lead to greater errors, potentially interfering with the results. Additionally, due to differences in the active ingredients of each DSCIs, their effects vary among different types of patients. Clinically, the appropriate injection can be chosen based on the patient’s symptoms and signs to achieve better clinical efficacy. In reducing inflammatory markers, STS + WM had an advantage in lowering hs-CRP, DSDFSY + WM was more effective in reducing IL-1, and DSCXQ + WM was better at reducing IL-6. Regarding the impact on oxidative stress markers, DSDFSY + WM had a more significant effect on NO regulation, GXN + WM was more evident in SOD regulation, and DH + WM had an advantage in MDA regulation.

This study found that 49 RCTs included adverse reactions as an observed outcome, with 17 RCTs reporting specific adverse reactions. Clinical manifestations were mainly concentrated in the circulatory system, digestive system, peripheral vessels, and skin. The most common adverse reactions were dizziness, nausea, and palpitations (n = 17, n = 14, n = 12), followed by headache (n = 7), abdominal distension, rash, bruising (n = 6), facial flushing (n = 4), and fatigue (n = 3). The active ingredients in Danshen have vasodilatory effects (Deng et al., 2014; Lin et al., 2022), which may cause dizziness, headache, and facial flushing in users. These conditions are generally tolerable and can resolve with reduced infusion speed or discontinuation of the drug. Traditional Chinese medicine injections, containing large molecular metabolites, are prone to allergic reactions, such as rashes. Rapid infusion of multiple drugs can increase the incidence of allergic reactions (Zou et al., 2023) Therefore, we recommend the following precautions when using injections: ① Infusion speed should not be too fast and can be slowed down according to the patient’s age and tolerance. ② Do not combine with other injections, especially other traditional Chinese medicine injections. ③ Strictly follow the instructions, and do not arbitrarily change the dosage or frequency of administration. We recommend that future studies place greater emphasis on safety monitoring, including: ① Routine monitoring of liver and renal function, especially in patients with pre-existing conditions, due to the known effects of herbal treatments. ② Observation for allergic reactions, particularly during the initial stages of treatment. ③ Careful monitoring for bleeding risks, given the potential anticoagulant effects of Danshen injections, particularly when used alongside antiplatelet or anticoagulant drugs.

Danshen, a commonly used herbal medicine in TCM for the treatment of CHD, has a long history of clinical application due to its blood-activating and stasis-removing properties, which promote blood circulation and remove blood stasis. Danshen contains two major metabolites: hydrophilic phenolic acids and lipophilic tanshinones, both of which possess anti-oxidative stress, anti-inflammatory, and anti-thrombotic effects (Li et al., 2020; Ke et al., 2023). Among these, tanshinones (TSN) have potent cardiovascular protective effects (Li ZM. et al., 2018). TSN modulates multiple pathways to inhibit the development of atherosclerosis. For instance, study (Ma et al., 2023) has shown that Tanshinone IIA (Tan IIA) can reduce vascular endothelial inflammation and prevent plaque formation through the COX-2/TNF-α/NF-κB signaling pathway. Tanshinone I (Tan I) can inhibit oxidative stress and oxidative stress-induced cardiomyocyte damage by regulating the Nrf2 signaling pathway (Wu et al., 2021). Salvia acid A (SAA) inhibits TLR2/TLR4-mediated Myd88 activation and its downstream molecules TRAF6 and IRAK4, thereby reducing the release of pro-inflammatory cytokines and mediators (Dawuti et al., 2023). Additionally, research indicates that SAA significantly enhances the expression of Nrf2 and HO-1 in a dose-dependent manner, improving atherosclerosis (Song et al., 2019). Furthermore, study has demonstrated that intraperitoneal injection of Danshen in chronic iron overload mice can decrease MDA levels, increase SOD activity, and reverse oxidative stress-induced damage (Zhang Y. et al., 2015). DSCIS inhibits endothelial cell autophagy via the miR-19a/SIRT1 pathway, mitigating the effects of oxidative stress (Guo et al., 2021). Study has found that DSCIS reduces MDA levels and increases SOD activity in a dose-dependent manner, reducing oxidative stress (Du et al., 2021). Other research has shown that DSCIS has a disease-specific bidirectional regulatory effect on angiogenesis, promoting the repair of ischemic vascular injury through angiogenic activity while inhibiting tumor growth through anti-angiogenic activity (He et al., 2022). The observed differences in efficacy among the various DSCIs may be attributed to differences in their active ingredients, formulations, and mechanisms of action. However, the complex composition of traditional Chinese medicine injections warrants further investigation to fully understand the underlying mechanisms.

However, this study has several limitations. Firstly, the quality of the included literature is not high, with most RCTs not detailing the methods of random allocation and blinding, potentially leading to selection bias and detection bias, thus reducing the accuracy of the study. Secondly, there is heterogeneity in this study, which may be related to various factors such as different WM treatment methods, inclusion of populations at different stages of CHD, and varying doses and intervention times of DSCIs. Thirdly, the studies included are concentrated in different regions of China, and the efficacy in other countries and ethnic groups has not been evaluated, which may affect the generalizability of the results. Fourthly, this study focuses on the overall CHD population, and the recommendations for individualized treatment for patients at different stages of the disease are not sufficiently accurate. To reduce potential biases, we established transparent inclusion and exclusion criteria to ensure consistency across studies. For missing data, we applied multiple imputation techniques, and studies of low quality were excluded to improve the overall reliability of the results.

Therefore, we believe that to improve the accuracy of NMA results and provide more effective treatment recommendations, future clinical studies should adhere to RCT standard designs, improve methodological quality, and describe methodological key points in detail when publishing results to enhance the quality of evidence-based medicine. We hope future research directions that emphasize the need for larger, multicenter trials involving diverse populations across different countries and ethnic groups. This approach will enhance the robustness of our findings and contribute to a more comprehensive understanding of the studied phenomena. Despite certain limitations, the NMA analysis in this study evaluates the effects of different treatment regimens on various outcome indicators, providing recommendations for clinical treatment of CHD.

## 5 Conclusion

In summary, this study demonstrates that the combination of DSCIs and WM treatment is more effective than WM treatment alone for patients with CHD. This includes improvements in clinical symptoms, electrocardiogram efficacy evaluation, and hematological parameters (inflammatory markers and oxidative stress markers). Overall, DSDFSY and DSCXQ showed favorable performance in clinical efficacy evaluation and inflammatory marker modulation, while DH exhibited stable performance in oxidative stress regulation. Although this study partially confirmed the efficacy of DSCIs, there are still shortcomings in the level of evidence and clinical application. In the future, we hope to conduct rigorous, high-quality randomized controlled trials to further clarify the efficacy and mechanisms of DSCIs, thereby facilitating their clinical use.

## Abbreviations

DSCIs, Danshen class injection; CHD, coronary heart disease; NMA, network meta-analysis; RCT, randomized controlled trials; CNKI, China National Knowledge Infrastructure; VIP, China Science and Technology Journal Database; CBM, Chinese Biomedical Literature Database; CCS, chronic coronary syndrome; ACS, acute coronary syndrome; PCI, percutaneous coronary intervention; TCM, traditional Chinese medicine; TSN, tanshinones; Tan IIA, Tanshinone IIA; Tan I, Tanshinone I; SAA, Salvia acid A; WM, western medicine; DH, Danhong injection; DS, Danshen injection; DSCXQ, Danshenchuanxiongqin injection; DSDFSY, Dansenduofensuanyan injection; DSFZ, Danshenfen injection; FFDS, Fufang Danshen injection; GXN, Guanxinning injection; STS, Sodium Tanshinone IIA Sulfonate injection; XD, Xiangdan injection; SXPTT, Shenxiongputaotang injection. hs-CRP, high-sensitivity C-reactive protein; IL-1, interleukin-1; IL-6, interleukin-6; NO, nitric oxide; SOD, superoxide dismutase; MDA, malondialdehyde.
