# Supplementary material for: Efficacy and safety of danshen class injections in the treatment of coronary heart disease: a network meta-analysis
Source: Front Pharmacol. 2024 Dec 12;15:1487119. doi: 10.3389/fphar.2024.1487119 (PMC11669530; doi:10.3389/fphar.2024.1487119)
Supplement: Supplementary file 1 [file DataSheet1.docx]

**Supplementary Table 1. Risk of bias judgments for RCTs (RoB 2.0)**

| Study ID | Bias arising from the randomization process | Bias due to deviations from intended intervention | Bias due to missing outcome data | Bias in measurement of the outcome | Bias in selection of the reported result | Overall |
| --- | --- | --- | --- | --- | --- | --- |
| Guo G.L._2016^[34]^ | Some concerns | Low | Low | Some concerns | Low | Some concerns |
| Lv X.S._2018^[35]^ | Some concerns | Low | Low | Some concerns | Low | Some concerns |
| Wu X.L._2010^[36]^ | Some concerns | Low | Low | Some concerns | Low | Some concerns |
| Zhang C.J._2018^[37]^ | Some concerns | Low | Low | Some concerns | Low | Some concerns |
| Qiu C.Z._2017^[38]^ | Some concerns | Low | Low | Some concerns | Low | Some concerns |
| Xi H.W._2015^[39]^ | Some concerns | Low | Low | Some concerns | Low | Some concerns |
| Bi C.W._2011^[40]^ | Some concerns | Low | Low | Some concerns | Low | Some concerns |
| Song G.M._2015^[41]^ | Some concerns | Low | Low | Some concerns | Low | Some concerns |
| Liu J.Z._2015^[42]^ | Some concerns | Low | Low | Some concerns | Low | Some concerns |
| Cai L.L._2014^[43]^ | Some concerns | Low | Low | Some concerns | Low | Some concerns |
| Li Y.F._2012^[44]^ | Some concerns | Low | Low | Some concerns | Low | Some concerns |
| Yu M.H._2016^[45]^ | Some concerns | Low | Low | Some concerns | Some concerns | Some concerns |
| Ji S.L._2013^[46]^ | Some concerns | Low | Low | Some concerns | Some concerns | Some concerns |
| Liu G.D._2014^[47]^ | Some concerns | Low | Low | Some concerns | Low | Some concerns |
| Chen S.L._2016^[48]^ | Some concerns | Low | Low | Some concerns | Low | Some concerns |
| Wang N._2017^[49]^ | Some concerns | Low | Low | Some concerns | Low | Some concerns |
| Mao Y.Y._2016^[50]^ | Some concerns | Low | Low | Some concerns | Some concerns | Some concerns |
| Jiang Z.P._2012^[51]^ | Some concerns | Low | Low | Some concerns | Some concerns | Some concerns |
| Tao L._2014^[52]^ | Some concerns | Low | Low | Some concerns | Some concerns | Some concerns |
| Zhang W._2015^[53]^ | Some concerns | Low | Low | Some concerns | Low | Some concerns |
| Li X._2018^[54]^ | Some concerns | Low | Low | Some concerns | Low | Some concerns |
| Zhu Q.K._2019^[55]^ | Some concerns | Low | Low | Some concerns | Some concerns | Some concerns |
| Liu L.L._2023^[56]^ | Some concerns | Low | Low | Some concerns | Low | Some concerns |
| Jiang X._2022^[57]^ | Some concerns | Low | Low | Some concerns | Low | Some concerns |
| Jing L.X._2014^[58]^ | Some concerns | Low | Low | Some concerns | Low | Some concerns |
| Xia L._2022^[59]^ | Some concerns | Low | Low | Some concerns | Low | Some concerns |
| Jiao W.P._2019^[60]^ | Some concerns | Low | Low | Some concerns | Low | Some concerns |
| Pan D.M._2018^[61]^ | Some concerns | Low | Low | Some concerns | Low | Some concerns |
| Qiu Y.H._2020^[62]^ | Some concerns | Low | Low | Some concerns | Low | Some concerns |
| Xie Q.T._2020^[63]^ | Some concerns | Low | Low | Some concerns | Low | Some concerns |
| Yue H.J._2023^[64]^ | Some concerns | Low | Low | Some concerns | Low | Some concerns |
| Cheng J.L._2012^[65]^ | Some concerns | Low | Low | Some concerns | Low | Some concerns |
| Yang N._2010^[66]^ | Some concerns | Low | Low | Some concerns | Low | Some concerns |
| Li Y.M._2007^[67]^ | Some concerns | Low | Low | Some concerns | Some concerns | Some concerns |
| Li H._2008^[68]^ | Some concerns | Low | Low | Some concerns | Low | Some concerns |
| Huang D.P._2017^[69]^ | Some concerns | Low | Low | Some concerns | Low | Some concerns |
| Xie S._2011^[70]^ | Some concerns | Low | Low | Some concerns | Low | Some concerns |
| Mei F.G._2006^[71]^ | Some concerns | Low | Low | Some concerns | Low | Some concerns |
| Liao Y.H._2008^[72]^ | Some concerns | Low | Low | Some concerns | Low | Some concerns |
| Zhao B._2015^[73]^ | Some concerns | Low | Low | Some concerns | Low | Some concerns |
| Zhang B.H._2011^[74]^ | Some concerns | Low | Low | Some concerns | Low | Some concerns |
| Yang C.P._2009^[75]^ | Some concerns | Low | Low | Some concerns | Low | Some concerns |
| Peng H.S._2015^[76]^ | Some concerns | Low | Low | Some concerns | Low | Some concerns |
| Fu T._2015^[77]^ | Some concerns | Low | Low | Some concerns | Low | Some concerns |
| Wan S.J._2020^[78]^ | Some concerns | Low | Low | Some concerns | Low | Some concerns |
| Li C._2008^[79]^ | Some concerns | Low | Low | Some concerns | Low | Some concerns |
| Zhao H._2011^[80]^ | Some concerns | Low | Low | Some concerns | Some concerns | Some concerns |
| Chen S.Q._2015^[81]^ | Some concerns | Low | Low | Some concerns | Low | Some concerns |
| Liu Y.F._2012^[82]^ | Some concerns | Low | Low | Some concerns | Low | Some concerns |
| Li Y.H._2011^[83]^ | Some concerns | Low | Low | Some concerns | Low | Some concerns |
| Shi Y.P._2012^[84]^ | Some concerns | Low | Low | Some concerns | Low | Some concerns |
| Chen J.H._2015^[85]^ | Some concerns | Low | Low | Some concerns | Low | Some concerns |
| Xu L.J._2014^[86]^ | Some concerns | Low | Low | Some concerns | Low | Some concerns |
| Fu Y.C._2011^[87]^ | Some concerns | Low | Low | Some concerns | Low | Some concerns |
| Zhang S.G._2012^[88]^ | Some concerns | Low | Low | Some concerns | Low | Some concerns |
| Zhang X.Y._2016^[89]^ | Some concerns | Low | Low | Some concerns | Low | Some concerns |
| Hong Y.D._2004^[90]^ | Some concerns | Low | Low | Some concerns | Low | Some concerns |
| Lang X.F._2012^[91]^ | Some concerns | Low | Low | Some concerns | Low | Some concerns |
| Yu M.K._2014^[92]^ | Some concerns | Low | Low | Some concerns | Low | Some concerns |
| Lu J._2023^[93]^ | Some concerns | Low | Low | Some concerns | Low | Some concerns |
| Feng Z.H._2010^[94]^ | Some concerns | Low | Low | Some concerns | Low | Some concerns |
| Mei F.G._2014^[95]^ | Some concerns | Low | Low | Some concerns | Low | Some concerns |
| Xu Z.J._2022^[96]^ | Some concerns | Low | Low | Some concerns | Low | Some concerns |
| Chen S.L._2016^[97]^ | Some concerns | Low | Low | Some concerns | Low | Some concerns |
| Zhang R.H._2010^[98]^ | Some concerns | Low | Low | Some concerns | Low | Some concerns |
| Wu Z.M._2012^[99]^ | Some concerns | Low | Low | Some concerns | Low | Some concerns |
| Duan X.L._2010^[100]^ | Some concerns | Low | Low | Some concerns | Low | Some concerns |
| Guan J.S._2019^[101]^ | Some concerns | Low | Low | Some concerns | Low | Some concerns |
| Lin Q.W._2020^[102]^ | Some concerns | Low | Low | Some concerns | Low | Some concerns |
| Tan S.J._2009^[103]^ | Some concerns | Low | Low | Some concerns | Low | Some concerns |
| Pang Z._2014^[104]^ | Some concerns | Low | Low | Some concerns | Low | Some concerns |
| Liu M.Y._2017^[105]^ | Some concerns | Low | Low | Some concerns | Low | Some concerns |
| Ye T.H._2021^[106]^ | Some concerns | Low | Low | Some concerns | Low | Some concerns |
| Lun J._2011^[107]^ | Some concerns | Low | Low | Some concerns | Low | Some concerns |
| Qiu D._2018^[108]^ | Some concerns | Low | Low | Some concerns | Low | Some concerns |
| Chen F.L._2020^[109]^ | Some concerns | Low | Low | Some concerns | Low | Some concerns |
| Song J.J._2017^[110]^ | Some concerns | Low | Low | Some concerns | Low | Some concerns |
| Yang L.L._2010^[111]^ | Some concerns | Low | Low | Some concerns | Low | Some concerns |
| Zhu Y._2002^[112]^ | Some concerns | Low | Low | Some concerns | Low | Some concerns |
| Jin J._2012^[113]^ | Some concerns | Low | Low | Some concerns | Low | Some concerns |
| Wei Q._2014^[114]^ | Some concerns | Low | Low | Some concerns | Low | Some concerns |
| Fang Y._2018^[115]^ | Some concerns | Low | Low | Some concerns | Low | Some concerns |
| Mou L.N._2011^[116]^ | Some concerns | Low | Low | Some concerns | Low | Some concerns |
| Qi L.P._2001^[117]^ | Some concerns | Low | Low | Some concerns | Low | Some concerns |
| Hu A.Y._2011^[118]^ | Some concerns | Low | Low | Some concerns | Low | Some concerns |
| Luo X.C._2011^[119]^ | Some concerns | Low | Low | Some concerns | Low | Some concerns |
| Li H._2008^[120]^ | Some concerns | Low | Low | Some concerns | Low | Some concerns |
| Su X.M._2011^[121]^ | Some concerns | Low | Low | Some concerns | Low | Some concerns |
| Li H._2009^[122]^ | Some concerns | Low | Low | Some concerns | Low | Some concerns |
| Li H._2007^[123]^ | Some concerns | Low | Low | Some concerns | Low | Some concerns |
| Chen S._2009^[124]^ | Some concerns | Low | Low | Some concerns | Low | Some concerns |
| Liu J._2021^[125]^ | Low | Low | Low | Low | Low | Low |
| Li H.D._2018^[126]^ | Some concerns | Low | Low | Some concerns | Low | Some concerns |
| Sun L._2015^[127]^ | Some concerns | Low | Low | Some concerns | Low | Some concerns |
| Sun K._2014^[128]^ | Some concerns | Low | Low | Some concerns | Low | Some concerns |
| Li S.M._2014^[129]^ | Some concerns | Low | Low | Some concerns | Low | Some concerns |
| Yao H._2022^[130]^ | Some concerns | Low | Low | Some concerns | Low | Some concerns |
| Gao W._2009^[131]^ | Some concerns | Low | Low | Some concerns | Low | Some concerns |
| Chen Y.J._2021^[132]^ | Some concerns | Low | Low | Some concerns | Low | Some concerns |
| Zhang Z.J._2017^[133]^ | Some concerns | Low | Low | Some concerns | Low | Some concerns |
| Wang S._2009^[134]^ | Some concerns | Low | Low | Some concerns | Low | Some concerns |
| Liu B._2013^[135]^ | Some concerns | Low | Low | Some concerns | Low | Some concerns |
| Cao X.F._2013^[136]^ | Some concerns | Low | Low | Some concerns | Low | Some concerns |
| Yan C.G._2009^[137]^ | Some concerns | Low | Low | Some concerns | Low | Some concerns |
| Zhang L.L._2010^[138]^ | Some concerns | Low | Low | Some concerns | Low | Some concerns |
| Zhou S.N._2018^[139]^ | Some concerns | Low | Low | Some concerns | Low | Some concerns |

Note: Low: low risk of bias, there is the possibility of residual confounding that has not been controlled for (given the observational nature of the study), but otherwise little or no concern about bias in the result; Some concerns: there is some concern about bias with regard to this domain, although it is not clear that there is an important risk of bias; High: high risk of bias, the study has some important problems in this domain: characteristics of the study give risk to high risk of bias; Very high: very high risk of bias: the study is very problematic in this domain: characteristics of the study give rise to a very high risk of bias.

**Supplementary Table 2. The details of search terms and literature search strategy**

Take searching English databases as an example, the search terms and strategies are as follows:

**Pubmed**

#1 Coronary Disease”[Mesh]

#2 (((((((((((((((((Coronary Disease[Title/Abstract]) OR (Coronary Diseases[Title/Abstract])) OR (Disease, Coronary[Title/Abstract])) OR (Diseases, Coronary[Title/Abstract])) OR (Coronary Heart Disease[Title/Abstract])) OR (CHD[Title/Abstract])) OR (Coronary Heart Diseases[Title/Abstract])) OR (Disease, Coronary Heart[Title/Abstract])) OR (Diseases, Coronary Heart[Title/Abstract])) OR (Heart Disease, Coronary[Title/Abstract])) OR (Heart Diseases, Coronary[Title/Abstract])) OR (Coronary Artery Disease[Title/Abstract])) OR (CAD[Title/Abstract])) OR (Artery Disease, Coronary[Title/Abstract])) OR (Artery Diseases, Coronary[Title/Abstract])) OR (Coronary Artery Diseases[Title/Abstract])) OR (Disease, Coronary Artery[Title/Abstract])) OR (Diseases, Coronary Artery[Title/Abstract])

#3 #1 OR #2

#4 Salvia miltiorrhiza”[Mesh]

#5 (((((((((Danshen injection[Title/Abstract]) OR (Compound danshen injection[Title/Abstract])) OR (Xiangdan injection[Title/Abstract])) OR (Fufangdanshen injection[Title/Abstract])) OR (Danhong injection[Title/Abstract])) OR (Salvianolate injection[Title/Abstract])) OR (Salvianolic acid injection[Title/Abstract])) OR (Danshenchuanxiongqin injection[Title/Abstract])) OR (Sodium tanshinone IIA sulfonate injection[Title/Abstract])) OR (Guanxinning injection[Title/Abstract])

#6 #4 OR #5

#7 randomized controlled trial[Publication Type] OR randomized[Title/Abstract] OR placebo[Title/Abstract]

#8 #3 AND #6 AND #7

**Cochrane Library**

#1 Coronary Disease

#2 (Coronary Disease):ab,ti,kw OR (Coronary Diseases):ab,ti,kw OR (Disease, Coronary ):ab,ti,kw OR (Diseases, Coronary ):ab,ti,kw OR (Coronary Heart Disease ):ab,ti,kw OR (CHD):ab,ti,kw OR ( Coronary Heart Diseases):ab,ti,kw OR (Disease, Coronary Heart ):ab,ti,kw OR (Diseases, Coronary Heart):ab,ti,kw OR (Heart Disease, Coronary ):ab,ti,kw OR (Heart Diseases, Coronary):ab,ti,kw OR (Coronary Artery Disease):ab,ti,kw OR (CAD):ab,ti,kw OR (Artery Disease, Coronary):ab,ti,kw OR (Artery Diseases, Coronary):ab,ti,kw OR (Coronary Artery Diseases):ab,ti,kw OR (Disease, Coronary Artery ):ab,ti,kw OR (Diseases, Coronary Artery):ab,ti,kw

#3 #1 OR#2

#4 Salvia miltiorrhiza

#5 (Danshen injection):ab,ti,kw OR (Compound danshen injection):ab,ti,kw OR (Xiangdan injection):ab,ti,kw OR (Fufangdanshen injection):ab,ti,kw OR (Danhong injection):ab,ti,kw OR (Salvianolate injection):ab,ti,kw OR (Salvianolic acid injection):ab,ti,kw OR (Danshenchuanxiongqin injection):ab,ti,kw OR (Sodium tanshinone IIA sulfonate injection):ab,ti,kw OR (Guanxinning injection):ab,ti,kw

#6 #4 OR #5

#7 (randomized controlled trial):ab,ti,kw OR (randomized):ab,ti,kw OR (placebo):ab,ti,kw

#8 #3 AND #6 AND #7

**Embase**

#1 coronary AND ('disease'/exp OR disease)

#2 'Coronary Disease':ab,ti OR 'Coronary Diseases':ab,ti OR 'Disease, Coronary ':ab,ti OR 'Diseases, Coronary ':ab,ti OR 'Coronary Heart Disease ':ab,ti OR 'CHD':ab,ti OR ' Coronary Heart Diseases':ab,ti OR 'Disease, Coronary Heart ':ab,ti OR 'Diseases, Coronary Heart':ab,ti OR 'Heart Disease, Coronary ':ab,ti OR 'Heart Diseases, Coronary':ab,ti OR 'Coronary Artery Disease':ab,ti OR 'CAD':ab,ti OR 'Artery Disease, Coronary':ab,ti OR 'Artery Diseases, Coronary':ab,ti OR 'Coronary Artery Diseases':ab,ti OR 'Disease, Coronary Artery ':ab,ti OR 'Diseases, Coronary Artery':ab,ti

#3 #1 OR #2

#4 salvia AND miltiorrhiza

#5 'Danshen injection':ab,ti OR 'Compound danshen injection':ab,ti OR 'Xiangdan injection':ab,ti OR 'Fufangdanshen injection':ab,ti OR 'Danhong injection':ab,ti OR 'Salvianolate injection':ab,ti OR 'Salvianolic acid injection':ab,ti OR 'Danshenchuanxiongqin injection':ab,ti OR 'Sodium tanshinone IIA sulfonate injection':ab,ti OR 'Guanxinning injection':ab,ti

#6 #4 OR #5

#7 'randomized controlled trial':ab,ti OR 'randomized':ab,ti OR 'placebo':ab,ti

#8 #3 AND #6 AND #7

**Web of Science**

#1 TS=(Coronary Disease OR Coronary Diseases OR Disease, Coronary OR Diseases, Coronary OR Coronary Heart Disease OR CHD OR Coronary Heart Diseases OR Disease, Coronary Heart OR Diseases, Coronary Heart OR Heart Disease, Coronary OR Heart Diseases, Coronary OR Coronary Artery Disease OR CAD OR Artery Disease, Coronary OR Artery Diseases, Coronary OR Coronary Artery Diseases OR Disease, Coronary Artery OR Diseases, Coronary Artery)

#2 TS=(Danshen injection OR Compound danshen injection OR Xiangdan injection OR Fufangdanshen injection OR Danhong injection OR Salvianolate injection OR Salvianolic acid injection OR Danshenchuanxiongqin injection OR Sodium tanshinone IIA sulfonate injection OR Guanxinning injection)

#3 TS=(randomized controlled trial OR randomized OR placebo)

#4 #1 AND #2 AND #3

**Supplementary Table 3. Basic information on the ten kinds of SMICs to be included.**

| Number | Generic name | Chemical composition | Botanical/animal name |
| --- | --- | --- | --- |
| 1 | Danhong injection | Salvianolic acid A; Salvianolic acid B; Tanshinin sodium; Protocatechuic aldehyde; Caffeic acid; Rosemary acid | Salvia miltiorrhiza; Carthamus tinctorius |
| 2 | Danshen injection | salvia sodium; protocatechuic aldehyde; rosmarinic acid; salvianolic acid B | Salvia miltiorrhiza |
| 3 | Danshenchuanxiongqin injectio | Salvianol; Caffeic acid; Rosemary acid; Salvianolic acid; Kawarazin | Salvia miltiorrhiza; Ligusticum wallichii |
| 4 | Danshenduofensuanyan injection | salvianolic acid B | Salvia miltiorrhiza |
| 5 | Danshenfen injection | Salvianolic acids, tanshensu, rosmarinic acid, protocatechol | Salvia miltiorrhiza |
| 6 | Fufang Danshen  injection | Phenolic acids; Tanshinone IIA; Cryptotanshinone; Flavonoids; Dihydroflavonoids; Isoflavones | Salvia miltiorrhiza; Dalbergia odorifera |
| 7 | Guanxinning  injection | Salvianolic acids; Caffeoylquinic acid; Benzene phthalide; Organic acids; Amino acids; Sugar compounds; Protocatechualdehyde; Vanillin | Salvia miltiorrhiza; Ligusticum chuanxiong Hort |
| 8 | Sodium Tanshinone IIA Sulfonate injection | Tanshinone IIA | Salvia miltiorrhiza |
| 9 | Xiangdan injection | Tanshensin, protocatechuic acid, protocatechualdehyde, vanillic acid, caffeic acid, purple oxalic acid, rosmarinic acid, salvianolic acid B, salvianolic acid A | Salvia miltiorrhiza; Dalbergia officinalis |
| 10 | Shenxiongputaotang injection | Ligustrazine hydrochloride；Protocatechualdehyd; Caffeic acid ;Danshensu; Lithospermic acid; Salvianolic acid D; Rosmarinic acid; Salvianolic acid A; Salvianolic acid B | Salvia miltiorrhiza; Ligusticum chuanxiong |

**Supplementary Figure 1. Funnel plots of hs-CRP.**


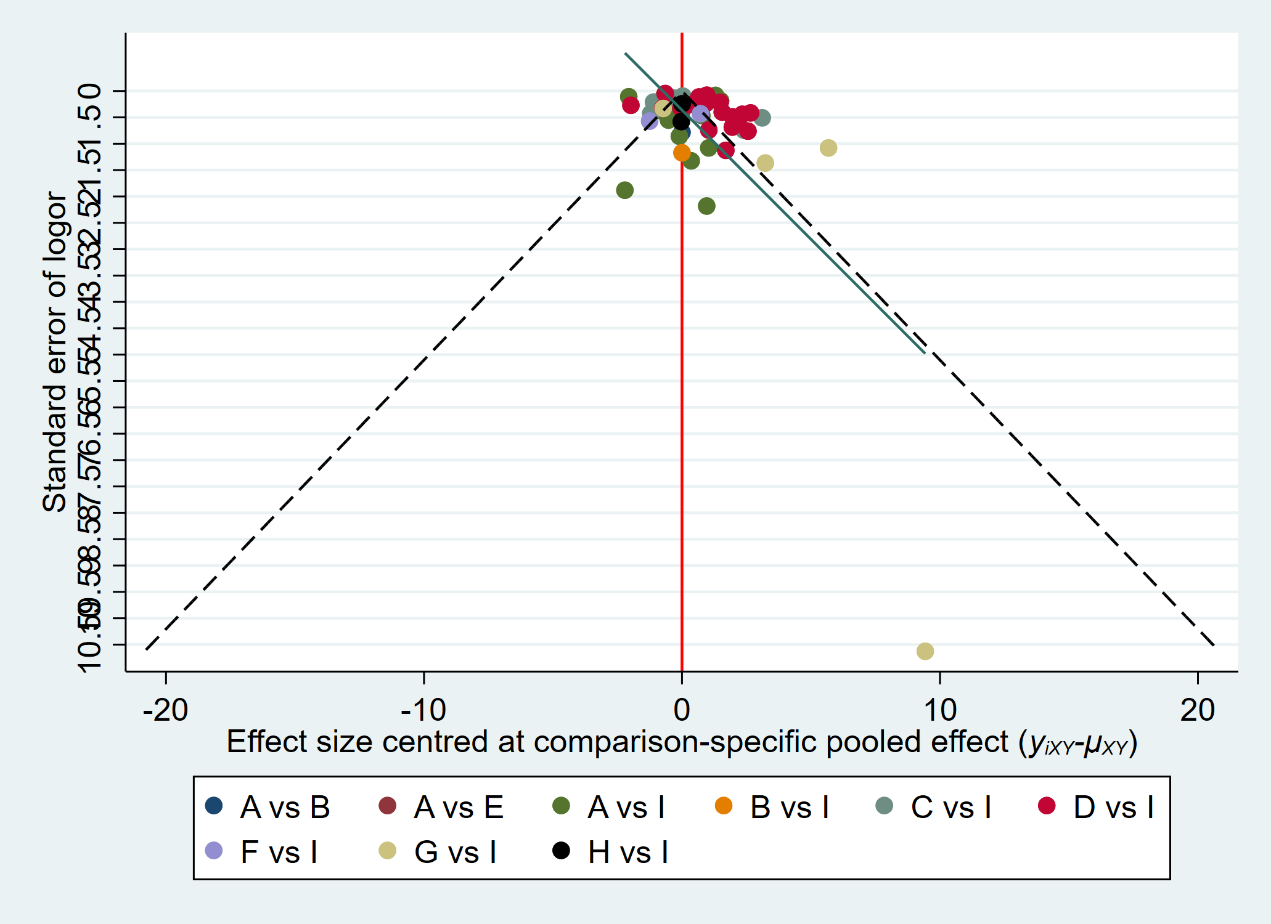


A, DH+WM; B, DS+WM; C, DSCXQ+WM; D, DSDFSY+WM; E, FFDS+WM; F, GXN+WM; G, STS+WM; H, SXPTT+WM; I, WM.

**Supplementary Figure 2. Funnel plots of IL-1.**


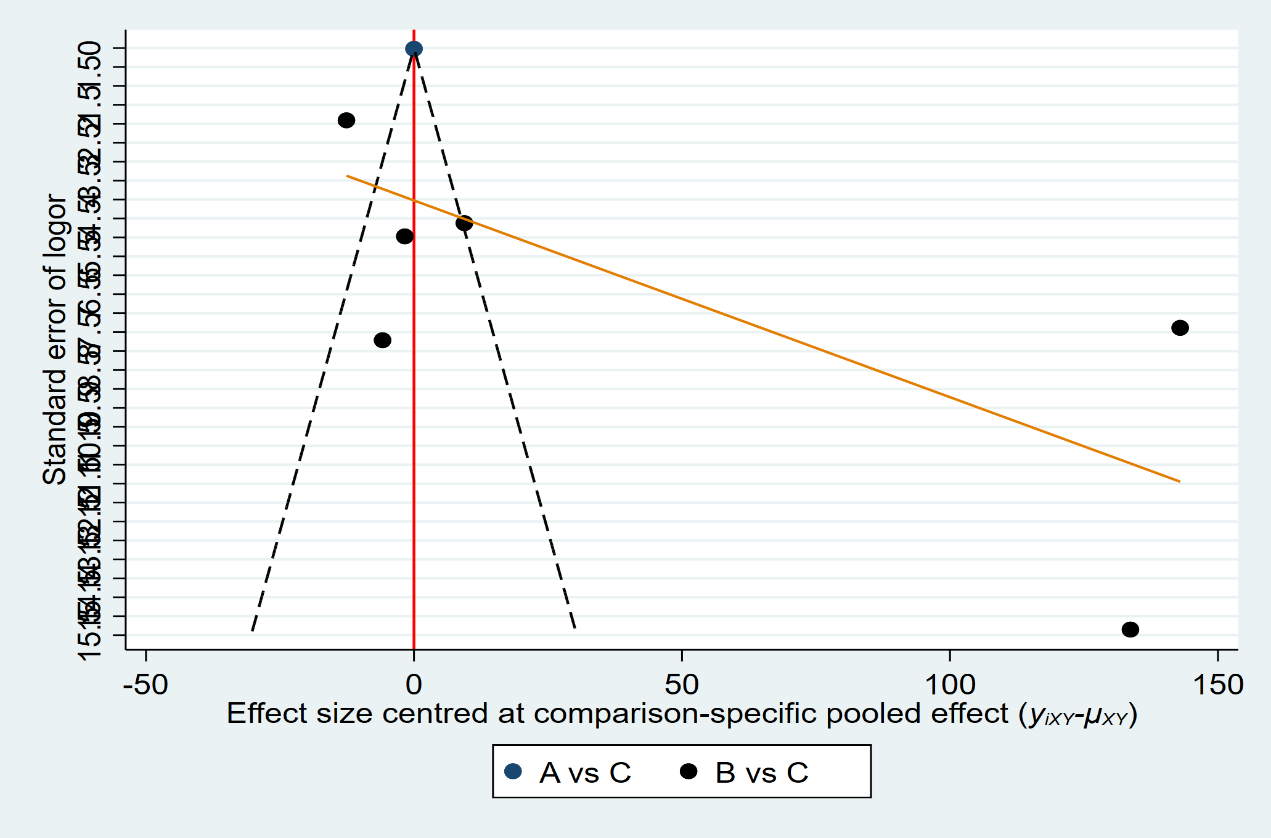


A, DH+WM; B, DSDFSY+WM; C, WM.

**Supplementary Figure 3. Funnel plots of IL-6.**


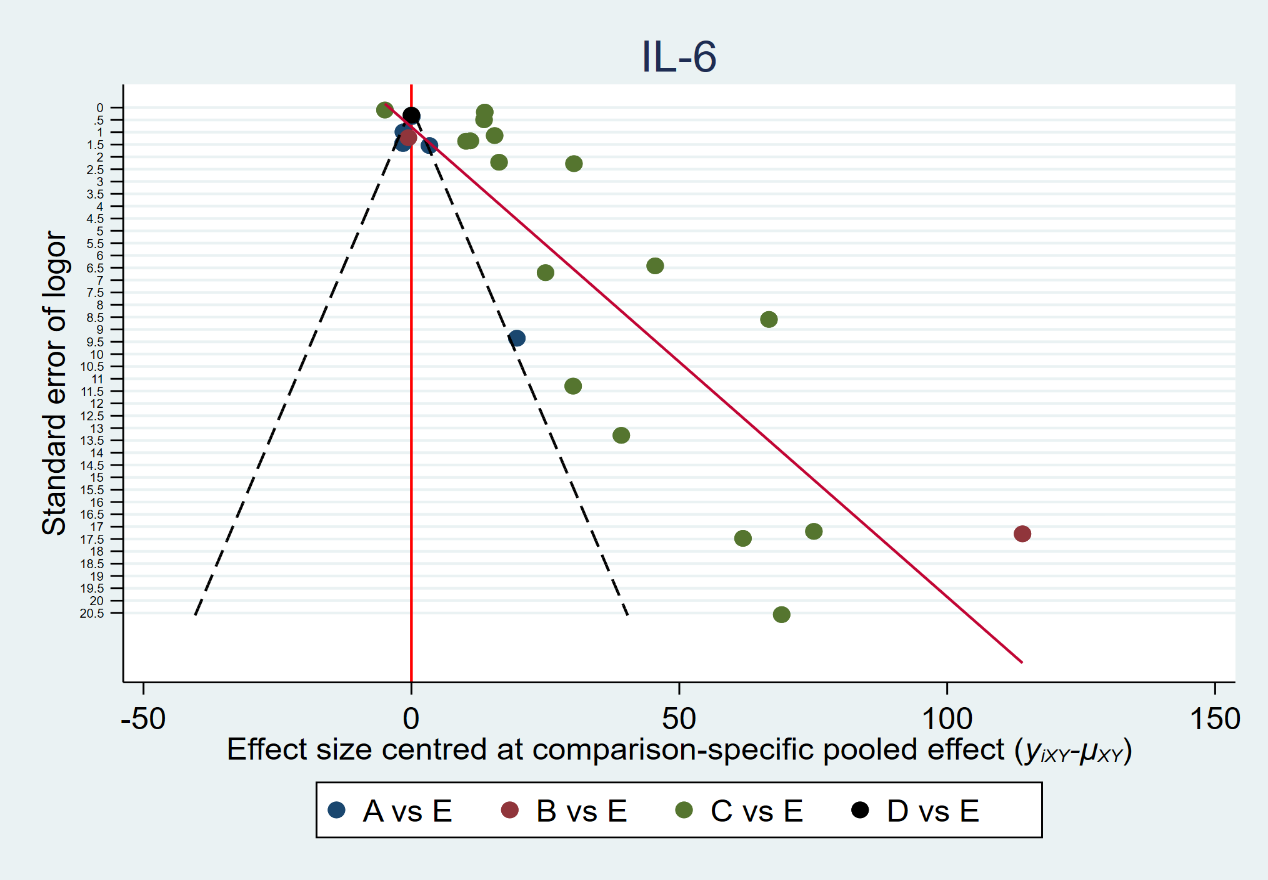


A, DH+WM; B, DSCXQ+WM; C, DSDFSY+WM; D, STS+WM; E, WM.

**Supplementary Figure 4. Funnel plots of NO.**


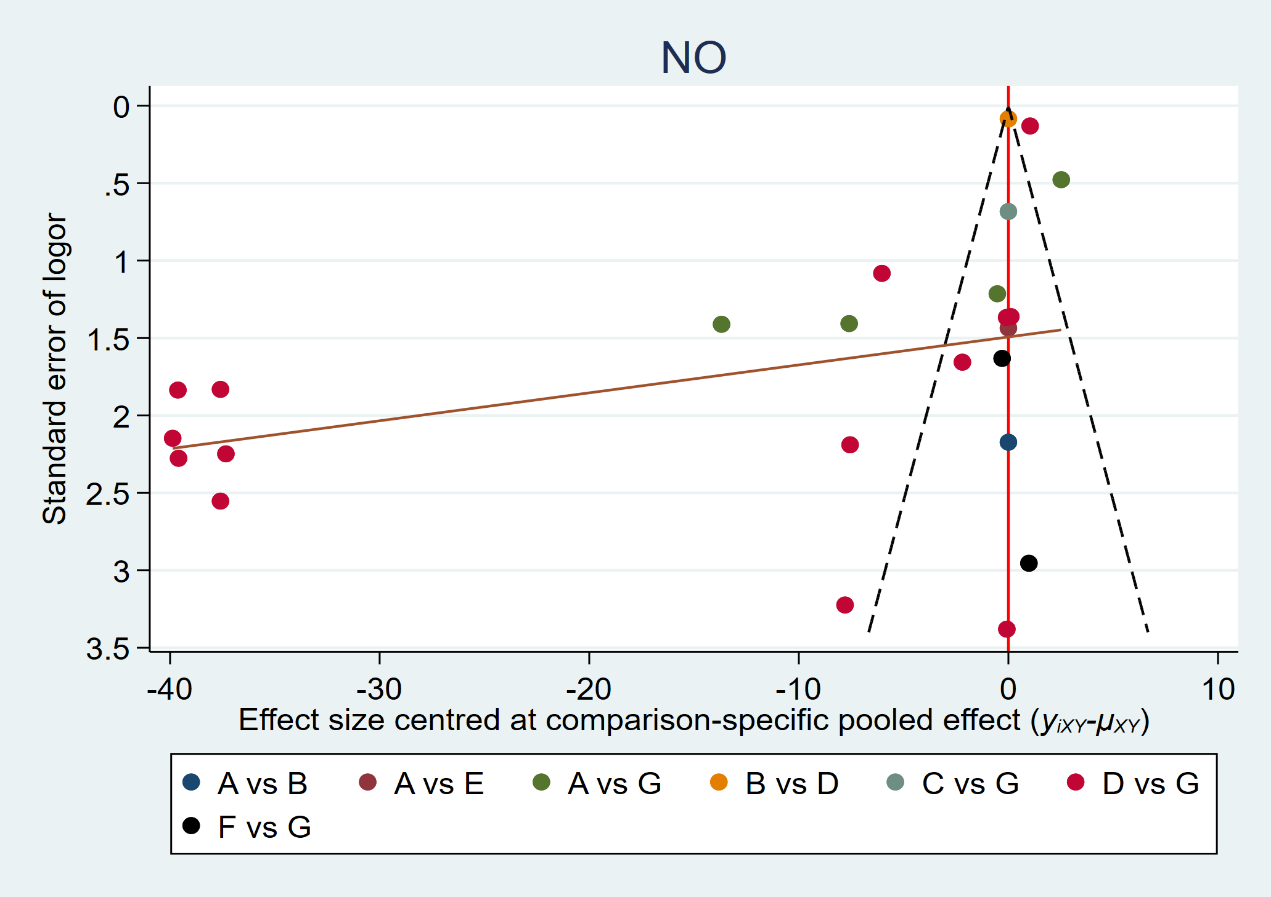


A, DH+WM; B, DS+WM; C, DSCXQ+WM; D, DSDFSY+WM; E, FFDS+WM; F, STS+WM; G, WM.

**Supplementary Figure 5. Funnel plots of SOD.**


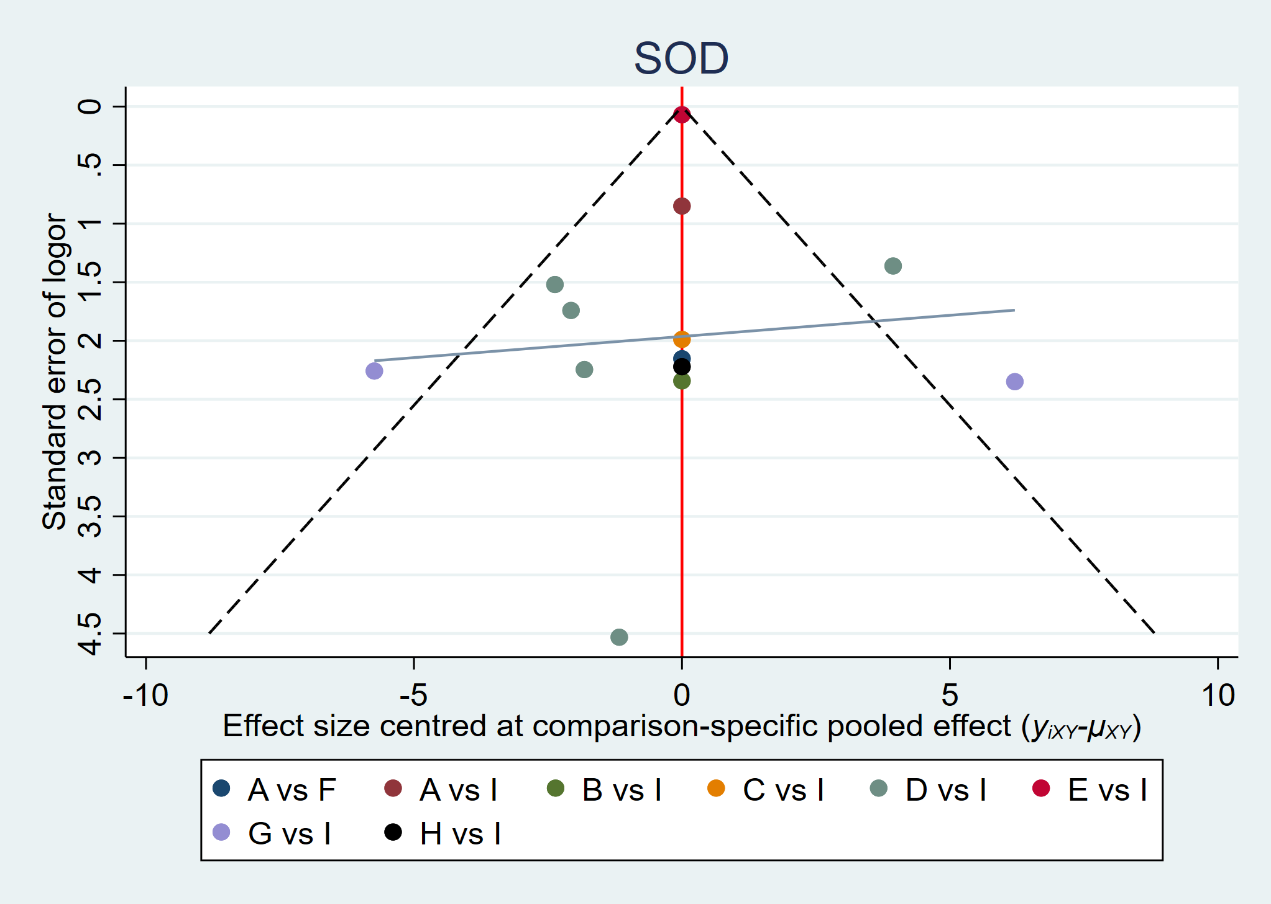


A, DH+WM; B, DS+WM; C, DSCXQ+WM; D, DSDFSY+WM; E, DSFZ+WM; F, FFDS+WM; G, GXN+WM; H, STS+WM; I, WM.

**Supplementary Figure 6. Funnel plots of MDA.**


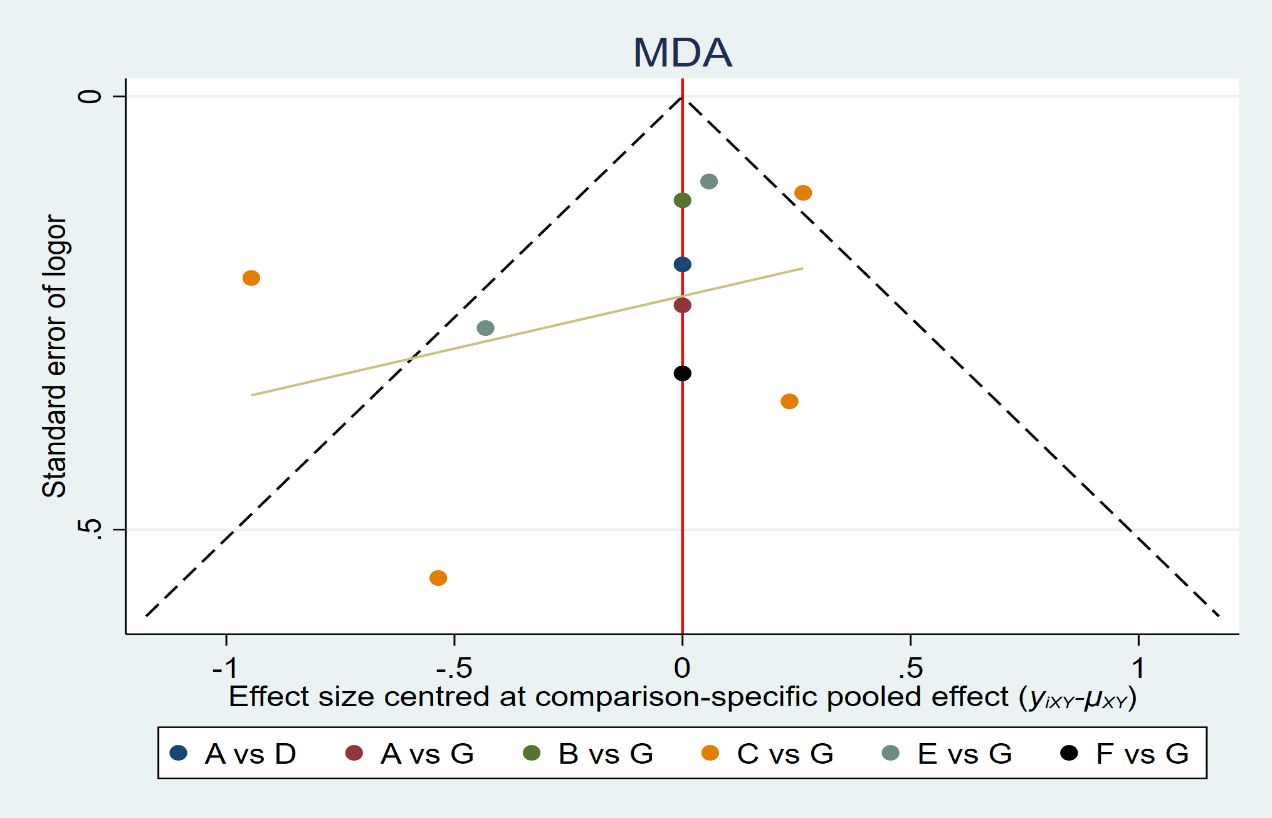


A, DH+WM; B, DS+WM; C, DSDFSY+WM; D, FFDS+WM; E, GXN+WM; F, STS+WM; G, WM.

**Supplementary Figure 7. Two-by-two comparison of clinical effectiveness rate**.


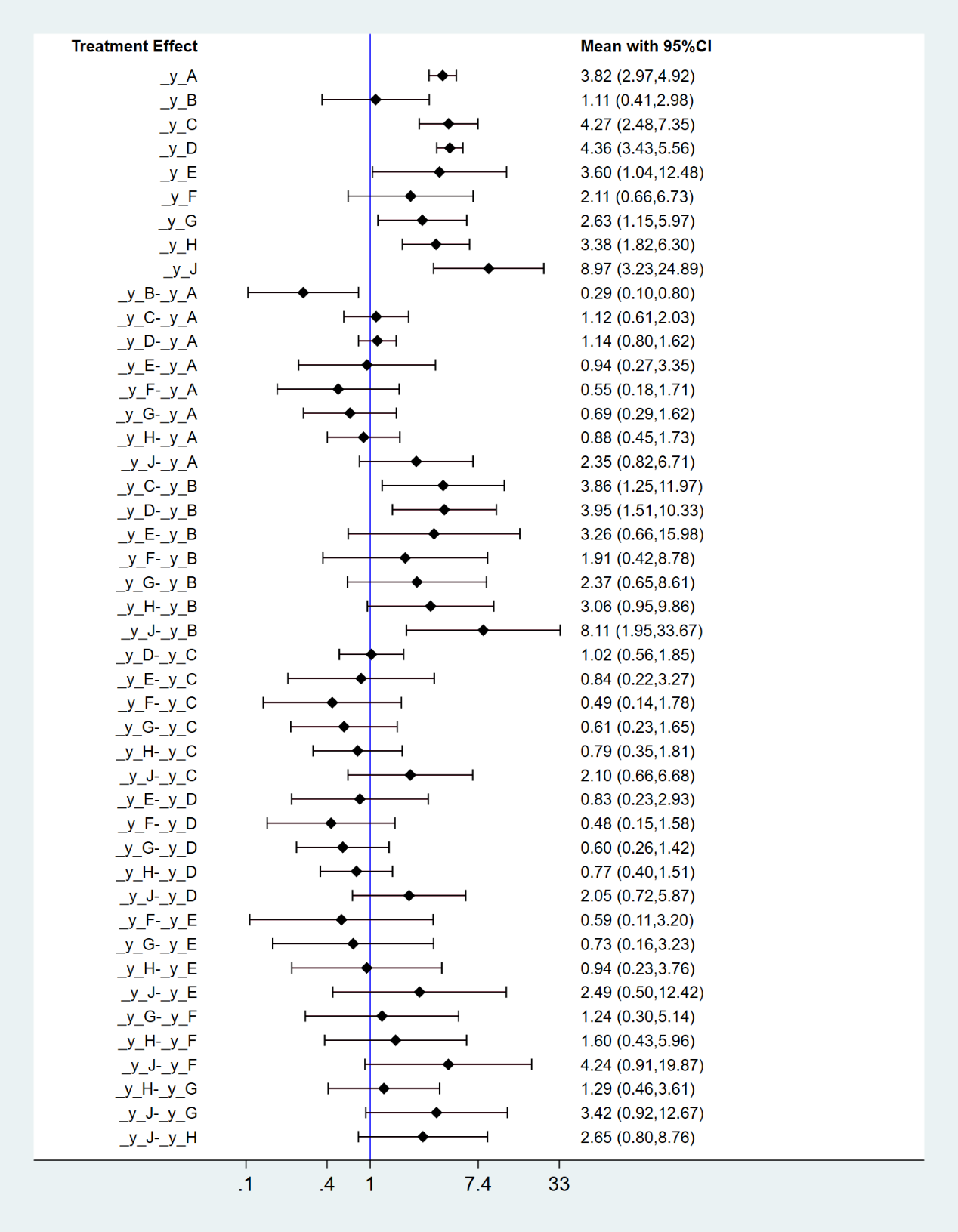


A, DH+WM; B, DS+WM; C, DSCXQ+WM; D, DSDFSY+WM; E, DSFZ+WM; F, FFDS+WM; G, GXN+WM; H, STS+WM; I, WM; J, XD+WM.

**Supplementary Figure 8. Two-by-two comparison of hs-CRP**.


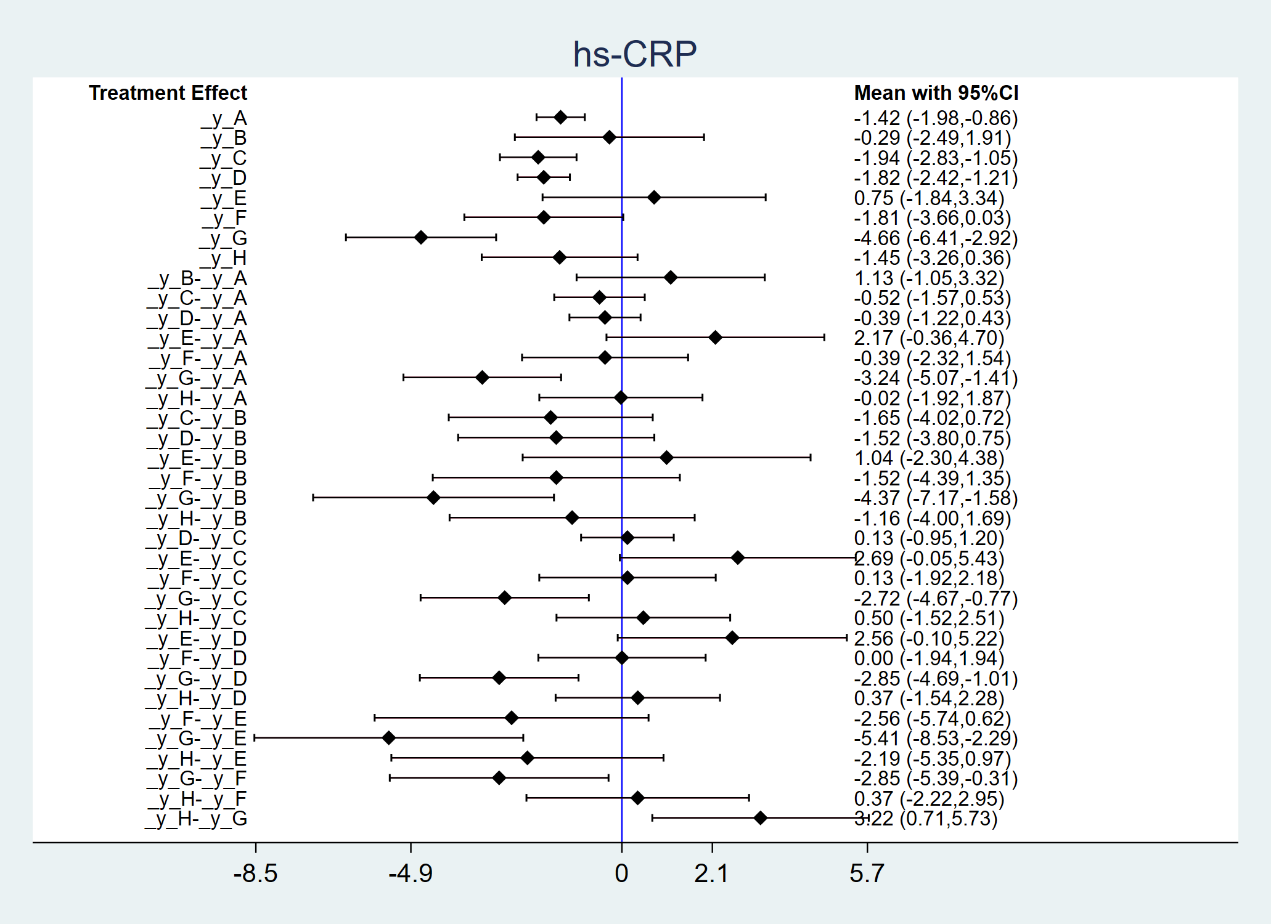


A, DH+WM; B, DS+WM; C, DSCXQ+WM; D, DSDFSY+WM; E, FFDS+WM; F, GXN+WM; G, STS+WM; H, SXPTT+WM; I, WM.

**Supplementary Figure 9. Two-by-two comparison of IL-1**.


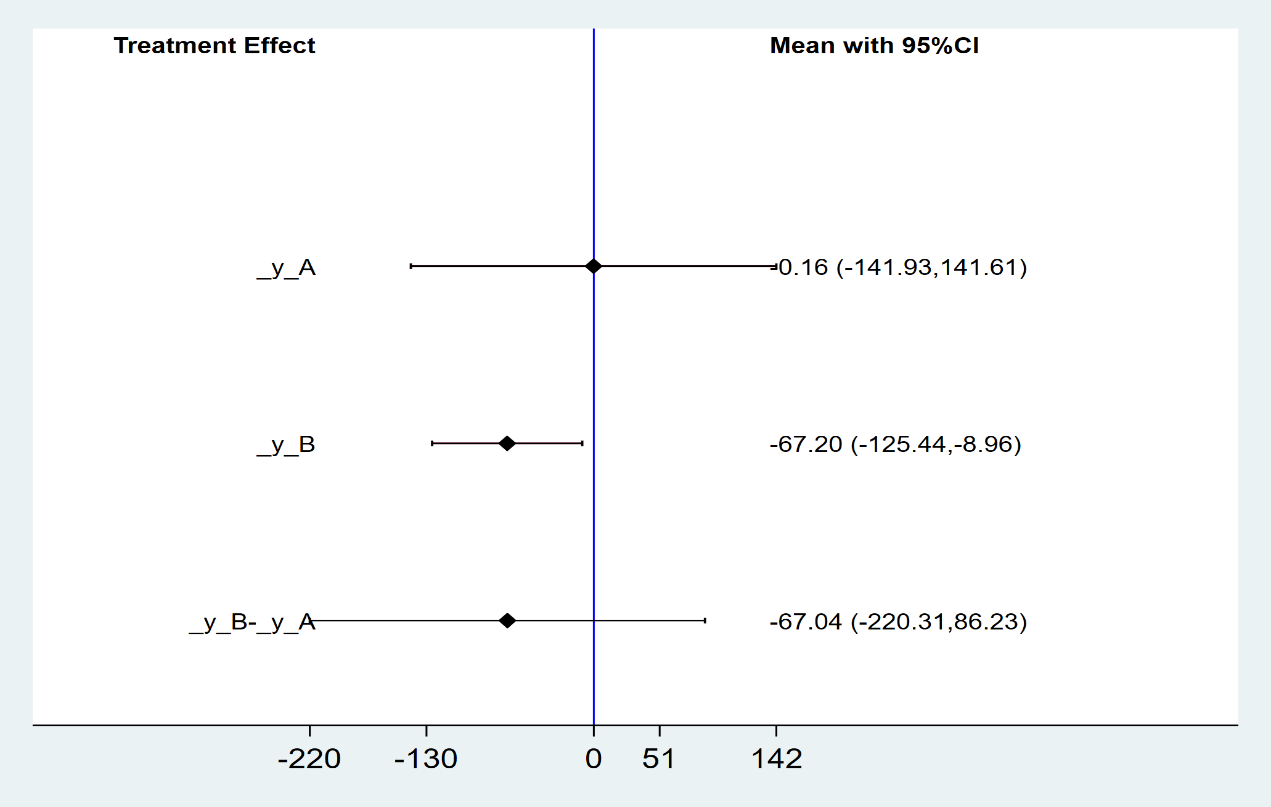


A, DH+WM; B, DSDFSY+WM; C, WM.

**Supplementary Figure 10. Two-by-two comparison of IL-6**.


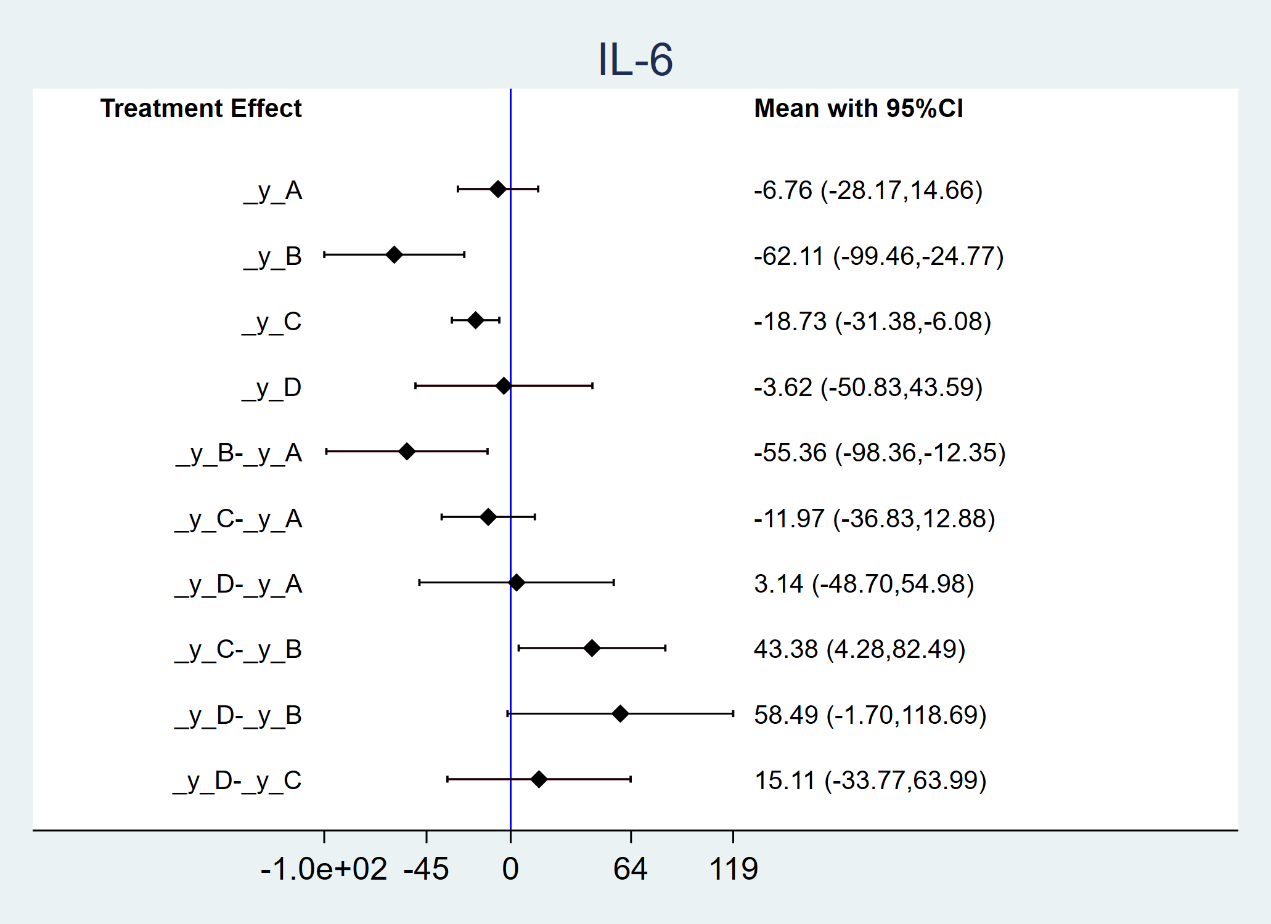


A, DH+WM; B, DSCXQ+WM; C, DSDFSY+WM; D, STS+WM; E, WM.

**Supplementary Figure 11. Two-by-two comparison of NO**.


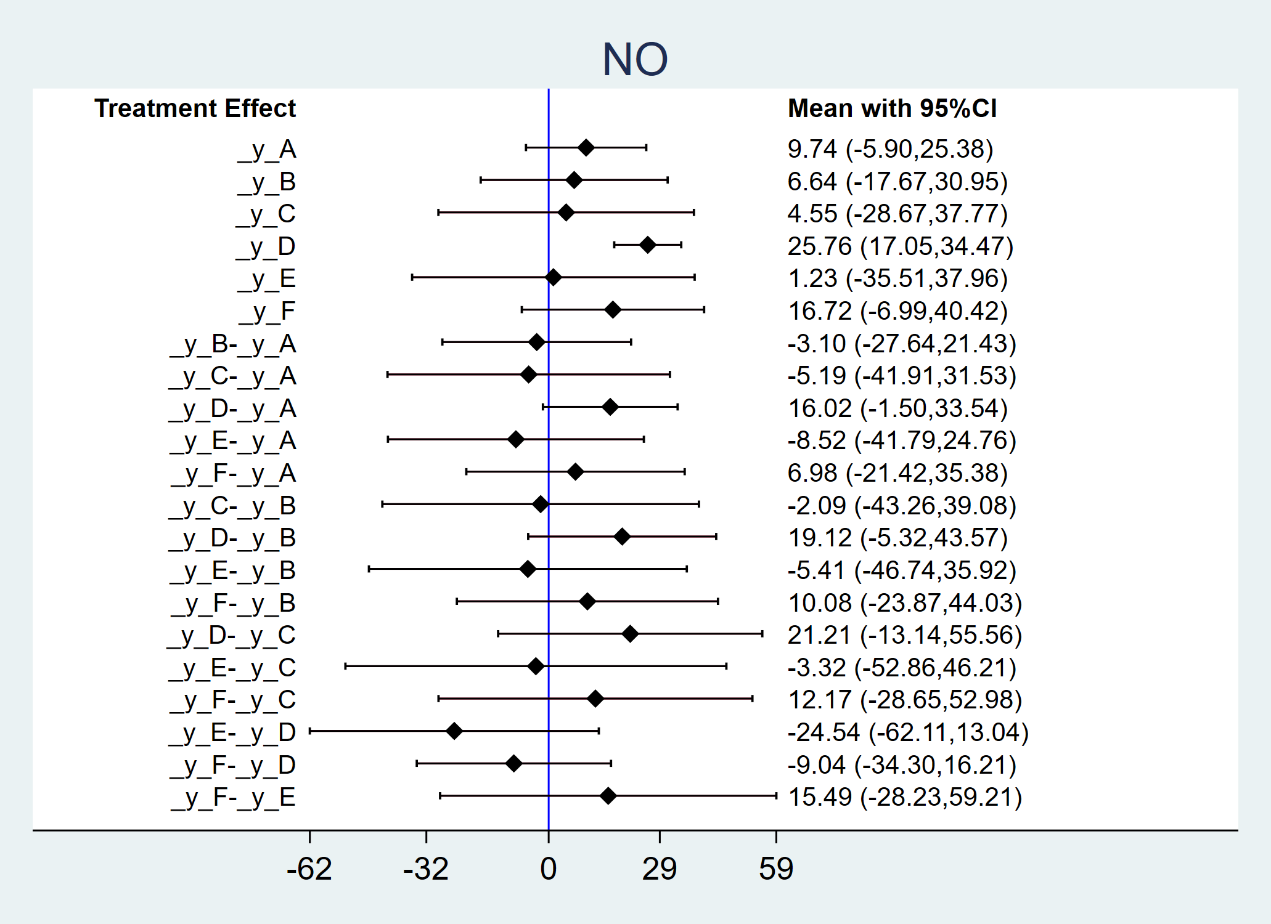


A, DH+WM; B, DS+WM; C, DSCXQ+WM; D, DSDFSY+WM; E, FFDS+WM; F, STS+WM; G, WM.

**Supplementary Figure 12. Two-by-two comparison of SOD**.


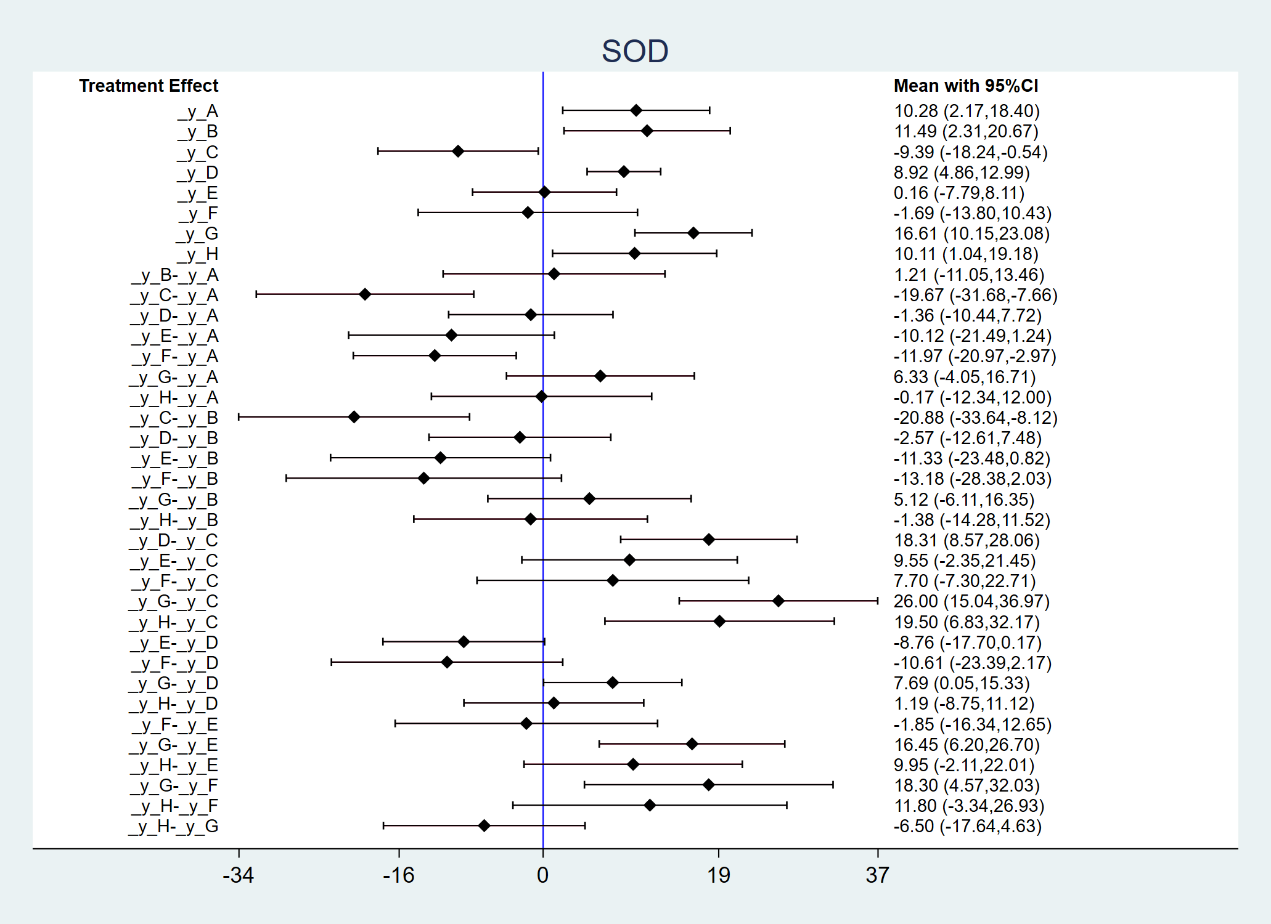


A, DH+WM; B, DS+WM; C, DSCXQ+WM; D, DSDFSY+WM; E, DSFZ+WM; F, FFDS+WM; G, GXN+WM; H, STS+WM; I, WM.

**Supplementary Figure 13. Two-by-two comparison of MDA**.


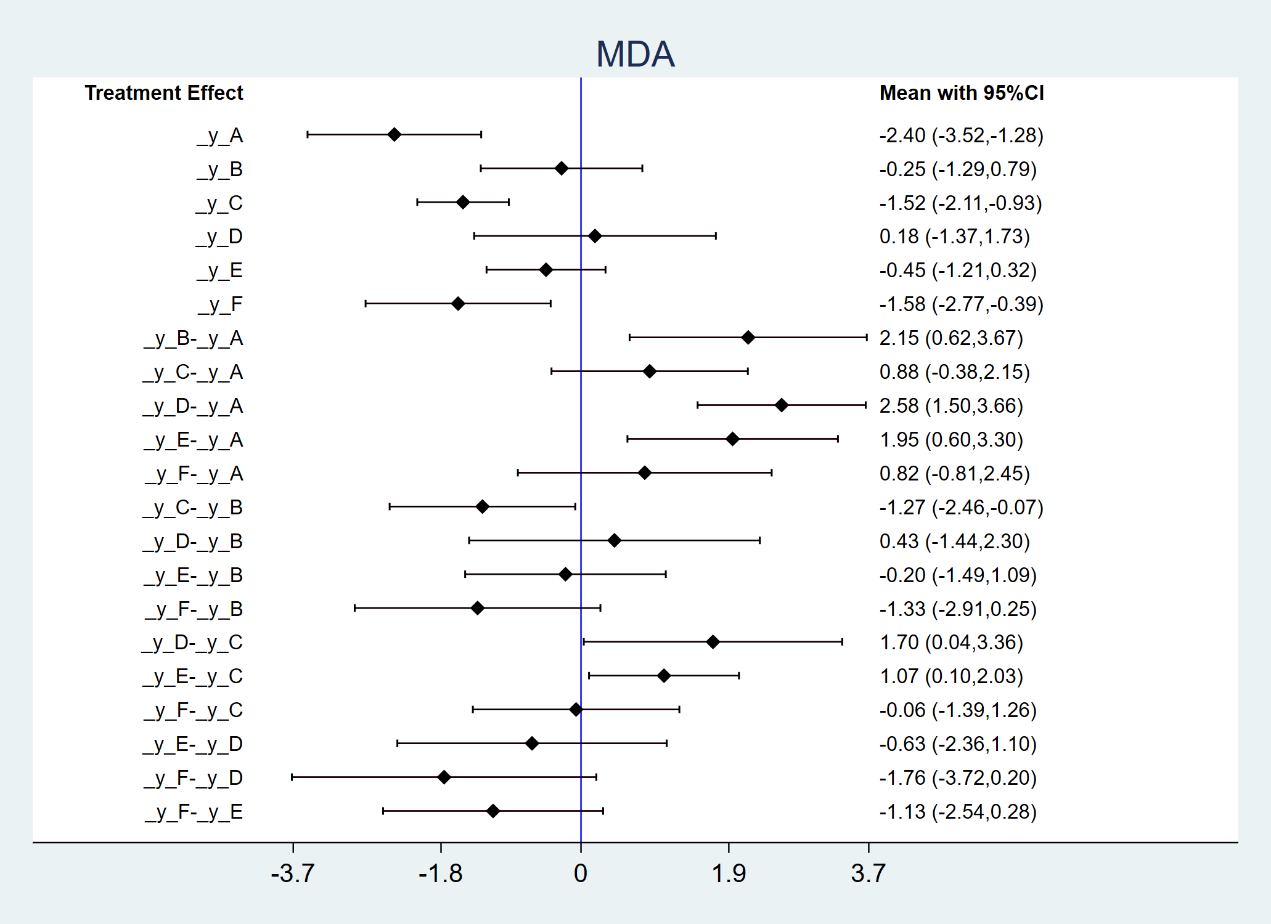


A, DH+WM; B, DS+WM; C, DSDFSY+WM; D, FFDS+WM; E, GXN+WM; F, STS+WM; G, WM.
